# Crude Oil Degradation in Temperatures Below the Freezing Point by Bacteria from Hydrocarbon-Contaminated Arctic Soils and the Genome Analysis of *Sphingomonas* sp. AR_OL41

**DOI:** 10.3390/microorganisms12010079

**Published:** 2023-12-30

**Authors:** Ekaterina M. Semenova, Tatyana P. Tourova, Tamara L. Babich, Ekaterina Y. Logvinova, Diyana S. Sokolova, Nataliya G. Loiko, Vladimir A. Myazin, Maria V. Korneykova, Andrey V. Mardanov, Tamara N. Nazina

**Affiliations:** 1 Winogradsky Institute of Microbiology, Research Center of Biotechnology, Russian Academy of Sciences, 119071 Moscow, Russia; semenova_inmi@mail.ru (E.M.S.); tptour@rambler.ru (T.P.T.); microb101@yandex.ru (T.L.B.); logvinovaekaterina@gmail.com (E.Y.L.); sokolovadiyana@gmail.com (D.S.S.); loikonat@mail.ru (N.G.L.); 2 Institute of North Industrial Ecology Problems–Subdivision of the Federal Research Centre “Kola Science Centre of Russian Academy of Science”, 184209 Apatity, Russia; korneykova.maria@mail.ru; 3 Agrarian and Technological Institute, People’s Friendship University of Russia (RUDN University), 117198 Moscow, Russia; 4 Institute of Bioengineering, Research Center of Biotechnology, Russian Academy of Sciences, 119071 Moscow, Russia; mardanov@biengi.ac.ru

**Keywords:** Arctic ground, psychrophiles, *n*-alkane oxidation, *Sphingomonas*, genome, *alkB* genes

## Abstract

Intensive human activity in the Arctic region leads to hydrocarbon pollution of reservoirs and soils. Isolation of bacteria capable of growing at low temperatures and degrading oil and petroleum products is of scientific and practical value. The aim of this work was to study the physiology and growth in oil at temperatures below 0 °C of four strains of bacteria of the genera *Pseudomonas*, *Rhodococcus*, *Arthrobacter*, and *Sphingomonas*—previously isolated from diesel-contaminated soils of the Franz Josef Land archipelago—as well as genomic analysis of the *Sphingomonas* sp. AR_OL41 strain. The studied strains grew on hydrocarbons at temperatures from −1.5 °C to 35 °C in the presence of 0–8% NaCl (*w*/*v*). Growth at a negative temperature was accompanied by visual changes in the size of cells as well as a narrowing of the spectrum of utilized *n*-alkanes. The studied strains were psychrotolerant, degraded natural biopolymers (xylan, chitin) and *n*-alkanes of petroleum, and converted phosphates into a soluble form. The ability to degrade *n*-alkanes is rare in members of the genus *Sphingomonas*. To understand how the *Sphingomonas* sp. AR_OL41 strain has adapted to a cold, diesel-contaminated environment, its genome was sequenced and analyzed. The Illumina HiSeq 2500 platform was used for AR_OL41 genome strain sequencing. The genome analysis of the AR_OL41 strain showed the presence of genes encoding enzymes of *n*-alkane oxidation, pyruvate metabolism, desaturation of membrane lipids, and the formation of exopolysaccharides, confirming the adaptation of the strain to hydrocarbon pollution and low habitat temperature. Average nucleotide identity and digital DNA–DNA hybridization values for genomes of the AR_OL41 strain with that of the phylogenetically relative *Sphingomonas alpine* DSM 22537^T^ strain were 81.9% and 20.9%, respectively, which allows the AR_OL41 strain to be assigned to a new species of the genus *Sphingomonas*. Phenomenological observations and genomic analysis indicate the possible participation of the studied strains in the self-purification of Arctic soils from hydrocarbons and their potential for biotechnological application in bioremediation of low-temperature environments.

## 1. Introduction

The Arctic region is rich in minerals (e.g., oil, gas, coal, metals); important shipping routes pass through it, which makes this region attractive for human activity despite the difficult climatic conditions [1]. Intensive development of the region has shown the vulnerability of natural ecosystems and their susceptibility to anthropogenic impact. Currently, the Arctic zone chronically suffers from the effects of human activity [2]. In addition to climate change, the development of the region has led to an increase in the number of man-made pollutants, which are very difficult to combat in northern conditions. Arctic soils are characterized by low organic matter content, which affects the sorption of hydrocarbons and their degradation. Permafrost restricts the movement of water, which can lead to the formation of a pollution zone in which most hydrocarbon pollutants are concentrated [3]. Imperfections of transport links and climatic features reduce the range of applied physicochemical methods of soil remediation [4,5]. Thus, the physical evacuation of contaminated permafrost soil is undesirable because this can contribute to thermokarst defrosting at the site of the removed soil layer via the formation of craters and sinkholes. Cleaning methods carried out directly on-site, such as bioremediation, can serve as an alternative solution to this problem. Bioremediation methods (biostimulation or bioaugmentation) are based on the ability of native bacteria [6,7,8,9] and yeasts [10,11] to degrade hydrocarbons. Soil moisture and temperature are the main parameters influencing the composition of soil microbial communities in the Arctic [12]. The number of bacterial cells in Arctic soils and soils can reach 10^6^–10^7^ cells/g [13,14]. Microorganisms that degrade oil and petroleum products have been found in frozen soils, snow, and sea ice [15,16,17,18,19,20,21]. Colla et al. [22,23] found no significant difference in soil microbial activity and oil degradation levels between biostimulation and bioaugmentation treatments. However, bioaugmentation allowed the removal of slightly more total petroleum hydrocarbon over the same time period. In laboratory experiments, the use of the sorption bioremediation method of soils of the Kola Peninsula based on the activation of native oil-oxidizing microorganisms by improving aerohydrodynamic conditions, the application of lime, mineral fertilizers, and granular coal allowed for a reduction in the content of total petroleum hydrocarbon by 78–91%, against 55% in soil without additives [24,25]. The introduction of additional psychrotolerant microorganisms can accelerate the purification process in case of poverty of the natural microbiome. The success of bioaugmentation depends on the adaptability of the introduced microorganisms to Arctic conditions [26]. Conducting further study of autochthonous bacteria in the self-purification of soils from petroleum products and the search for active oil destructors adapted to polar conditions is necessary to determine the best methods for soils bioremediation from hydrocarbon pollution in cold regions.

Previously, a number of bacterial strains of the genera *Pseudomonas*, *Rhodococcus*, *Arthrobacter*, and *Sphingomonas* using crude oil *n*-alkanes were isolated from diesel-contaminated soil samples taken on the island of Alexandra Land in the Franz Josef Land archipelago [27]. Bacteria of the genera *Pseudomonas* and *Sphingomonas* were also detected during metagenomic analysis of samples of ground soils and seawater, sea ice, and crude oil encapsulating the sea ice of the Arctic region [28,29]. The use of *n*-alkanes and other petroleum components has been shown for members of the genera *Pseudomonas*, *Rhodococcus*, and *Arthrobacter* [30,31,32,33,34], whereas for bacteria of the genus *Sphingomonas*, this property is rare [35,36,37,38]. In order to determine how well the isolated four strains are adapted to the habitat conditions, the growth of the strains in oil at low temperatures (9 and −1.5 °C) was studied, and physiological and metabolic properties (temperature and salinity ranges for growth, the spectrum of enzymes, the substrates used, differences from type strains of respective species) were determined. To find out how unique the ability to use *n*-alkanes is in bacteria of the *Sphingomonadaceae* family, it was necessary to sequence the genome of the *Sphingomonas* sp. AR_OL41 strain and compare it to the genomes available in GenBank for the presence of genes encoding the oxidation of *n*-alkanes as well as to analyze the cold stress protection genes that determine its adaptability to low temperatures. These data are necessary for the selection of bacteria for biotechnological applications for the remediation of soils contaminated with petroleum products in areas with low temperatures.

The purpose of this study was to determine the morphology, physiology, and biodegradation of oil at subfreezing temperatures for four strains of hydrocarbon-oxidizing bacteria of the genera *Pseudomonas*, *Rhodococcus*, *Arthrobacter*, and *Sphingomonas* isolated from Arctic soils as well as to analyze the genome and genes of *n*-alkanes biodegradation of the strain *Sphingomonas* sp. AR_OL 41.

## 2. Materials and Methods

### 2.1. Bacterial Strains

The objects of the study were four strains of aerobic hydrocarbon-oxidizing bacteria—*Pseudomonas frederiksbergensis* Ar-K7, *Rhodococcus yunnanensis* Ar-K9, *Arthrobacter alpinus* Ar-K10, and *Sphingomonas* sp. AR_OL41 (previously designated as AR-OL-41)—which were previously isolated from soil samples contaminated with diesel fuel on the Alexandra Land Island of the Franz Josef Land archipelago [27]. The strains Ar-K9, Ar-K10, and AR_OL41 were isolated from soil sampled at an altitude of 13.8 m above sea level (80°48′37″ N, 47°36′13″ E), and the Ar-K7 strain was isolated from soil sampled at an altitude of 12.8 m above sea level (80°48′46″ N, 47°33′52″ E). The *Sphingomonas* sp. AR_OL41 strain was deposited at the all-Russian collection of microorganisms (VKM; Pushchino, Moscow, Russia) under the number VKM B-3712^T^. 

### 2.2. Media Composition 

To maintain the studied strains and construct growth curves, TEG liquid medium containing the following (per liter of distilled water) was used: 5.0 g bacto-trypton, 2.5 g yeast extract, and 1.0 g glucose, pH 7.2 ± 0.2. Additionally, 20.0 g agar was added to obtain a solid medium. For strains Ar-K7, Ar-K9, and Ar-K10, 10 g of NaCl/L was added to the medium, and 1 g of NaCl/L was used for the AR_OL41 strain. In the study of growth at negative temperatures, all strains were incubated in the presence of 10 g NaCl per liter to prevent freezing of the medium. A solution of trace elements (1 mL/L) containing the following (mg per 100 mL distilled water) was added into all media: trilon B, 500; FeSO_4_·7H_2_O, 200; H_3_BO_4_, 30; CoCl_2_·6H_2_O, 20; ZnSO_4_·7H_2_O, 10; Na_2_MoO_4_·2H_2_O, 3; MnCl_2_·4H_2_O, 3; NiCl_2_·6H_2_O, 2; CuCl_2_·6H_2_O, 1 [39]. The range of substrates used (including oil) was tested on a mineral medium (MM) of the following composition (per liter distilled water): 1.5 g K_2_HPO_4_, 0.75 g KH_2_PO_4_, 1.0 g NH_4_Cl, 0.2 g MgSO_4_, 0.02 g CaCl_2_·2H_2_O, and 0.1 g KCl, pH 7.0 ± 0.2. Xylan, chitin, and microcrystalline cellulose (MCC) were used as substrates in concentrations (per liter) of 2.0 g, 1.0 g, and 1.0 g, respectively. Oil was added at a concentration of 0.3% (*v*/*w*). Moreover, 2,2,4,4,6,8,8-heptamethylnonane (5% *v*/*v*) was added to the oil as an internal standard for the degradation of *n*-alkanes. The bacteria were cultivated aerobically in stationary conditions for 7–30 days. The incubation temperature was indicated for each experiment.

Growth curves were constructed at 9 °C and 25 °C by cultivating bacteria in 1 L flasks under stationary conditions. Growth at various NaCl concentrations was studied at 22 °C. Growth at negative temperature (−1.5 °C) was studied on a TEG liquid medium with Teflon cubes for microbial fouling in stationary conditions for 14 days or on a mineral medium with oil for 30 days. For the scanning electron microscopy, biomass of the strains grown in a TEG medium with Teflon cubes at −1.5 °C and 9 °C was collected and prepared for the observation using phosphate buffer (pH 7.0), ethyl alcohol, and acetone, as described previously [40]. The dried preparations were mounted on special tables, and then a thin layer of metal (gold/palladium) was sprayed onto the preparations to create a conductive coating. The samples were examined under a scanning electron microscope (Quattro S, “Thermo Fisher Scientific Brno s.r.o.”, Brno-Černovice, Czech Republic) at an accelerating voltage of 10.0 kV, work distance of 9.4–10.1 mm, magnification of ×12,000–×65,000 at high vacuum mode.

Some biochemical properties of the isolated strains were determined using standard test kits, originally used for studies of gram-negative bacteria and later recommended for taxonomy studies of other prokaryotes [41]. Enzyme activity was detected using standard tests API^®^ Zym and API^®^ 20E (bioMérieux, Marcy l’Etoile, Auvergne-Rhône-Alpes, France) in accordance with the manufacturer’s instructions. Catalase activity was determined via the formation of gas bubbles after the addition of 3% (*v*/*v*) H_2_O_2_ to a freshly grown culture. Oxidase activity was determined using the oxidase reagent (bioMérieux, Marcy l’Etoile, Auvergne-Rhône-Alpes, France). The use of insoluble phosphates and their conversion to a soluble form was determined on a solid National Botanical Research Institute phosphate growth medium (NBRIP) that contained, per liter distilled water, 10.0 g glucose, 5.0 g Ca_3_(PO_4_)_2_, 5.0 g MgCl_2_·6H_2_O, 0.25 g MgSO_4_·7H_2_O, 0.2 g KCl, 0.1 g (NH_4_)_2_SO_4_, and 18.0 g agar, pH 7.0–7.2 [42]. The formation of an enlightenment zone around the colonies on the surface of the medium after 7 days of cultivation was considered a positive result. All experiments were performed in three repetitions. The results were statistically processed using Microsoft Excel, Microsoft Office 365 software.

### 2.3. A Model Experiment for Cleaning Sand from Oil

The Ar-K7, Ar-K9, and Ar-K10 strains were used in the experiments, in which 200 g of calcined quartz sand was introduced into a sterile plastic container; moistened with a sterile MM medium, 0.3% (*v*/*w*) sterile oil (with an internal standard); and a freshly grown culture was added to the final number of 10^7^ cells/g of sand. Previously, the number of cells in the culture used was estimated using the turbidity of the medium according to McFarland standards. The containers were thoroughly mixed, closed with sterile plastic lids, and incubated at 9 °C for 60 days. Once a week, the sand was mixed and, if necessary, moistened with sterile water. Sterile sand with oil without inoculum and sand with a culture without oil were used as controls. Every 30 days, changes in the number of microorganisms, the formation of lower fatty acids and alcohols, the formation of bacterial biofilms, and changes in the composition of the aliphatic fraction of oil were analyzed. After 60 days, the degradation of oil was determined by the weight method.

### 2.4. Analytical Methods

The growth of cultures was determined via changes in the turbidity of the medium compared to the control on an Ultrospec 2100 pro spectrophotometer (Amersham Biosciences, Slough, UK) at a wavelength of 660 nm and microscopy using an Axio Imager.D1 epifluorescence microscope (Carl Zeiss, Jena, Germany). Lower fatty acid and alcohol contents were determined using a GC-2010 Plus gas chromatograph (Shimadzu, Kyoto, Japan), as described earlier [43].

The utilization of oil was determined according to the change in the composition of the aliphatic fraction of oil compared to the control (in percent) using a Crystal 5000.1 gas-liquid chromatograph (Khromatek, Yoshkar-Ola, Russia) with a ZB-FFAP 15 m capillary column and a flame ionization detector, as described previously [44]. The use of oil in model experiments with sand was also determined via the weight method by extracting hydrocarbons with *n*-hexane in a Soxhlet extractor from a pre-dried sand sample (30 g) in a thermostat (75 °C). The extraction time was 1.5 h. The mass of oil was determined by the decrease in the mass of the sand sample after extraction and by the mass of petroleum products collected in the flask after evaporation of *n*-hexane. The mass fraction of petroleum products in the sand was calculated using the following formula:

X = (m_1_ − m_2_)100/m_0_

where X is the content of petroleum products, %; m_1_ is the mass of the sleeve with a sand attachment before extraction, g; m_2_ is the mass of the sleeve with a sand attachment after extraction, g; and m_0_ is the mass of the dehydrated sand attachment, g.

Biofilm formation was determined via staining using MTT (3-(4,5-dimethylthiazol-2-yl)-2,5-diphenyl-2H-tetrazolium bromide) and subsequent extraction of the formed formazane (H_2_NN=CHN=NH formic acid azohydrazone) using dimethyl sulfoxide [45,46]. The optical density of the extract was measured using an Ultrospec 2100 pro spectrophotometer (Amersham Biosciences, UK) at a wavelength of 540 nm.

### 2.5. DNA Extraction and 16S rRNA Gene and Genome Sequencing

DNA was isolated from the biomass of the *Sphingomonas* sp. AR_OL41 strain using a set of DiatomTM DNA Prep 100 reagents (IsoGen Inc., Moscow, Russia) according to the manufacturer’s recommendations. The purified DNA preparation was used as a matrix for PCR. Pure cultures were identified by 16S rRNA gene sequence analysis using the bacteria-universal primer set 27F–1492R [47]. DNA sequencing was performed according to the Sanger method on an ABI 3730 DNA analyzer automatic sequencer using the ABI PRISM^®^ BigDye™ Terminator v. 3.1 reagent kit (Applied Biosystems, Waltham, MA, USA). Taxonomic affiliation of the strains was determined using EzBioCloud [48].

Genomic DNA from the AR_OL41 strain was used for genome sequencing using the Illumina HiSeq 2500 platform (Illumina, Inc., San Diego, CA, USA). The library for Illumina sequencing was prepared using the NEBNext Ultra II DNA Library Prep Kit (New England Biolabs, Ipswich, MA, USA). Sequencing on the Illumina MiSeq generated 825,013 paired-end reads (2 × 300 nt, ~497 Mbp in total). Overlapping paired-end reads were merged using FLASH v.1.2.11 [49], and low-quality bases were trimmed using Sickle v.1.33 (https://github.com/najoshi/sickle; accessed on 28 January 2023) [50]. The reads were assembled de novo using SPAdes v. 3.15.4 software [51]. Default parameters were used for all software.

### 2.6. Bioinformatic Methods

The average nucleotide identity (ANI) was assessed using FastANI v. 1.34 (accessed on 29 July 2023) [52]. The digital DNA–DNA hybridization (dDDH) of genomes was performed using the Genome-to-Genome Distance Calculator v. 3.0 [53,54]. Identification of protein-coding sequences and primary annotation of genomes were performed using NCBI Prokaryotic Genome Automatic Annotation Pipeline, version 6.5, March 2023 [55]. To determine phylogenetically close taxa, we used the Type (Strain) Genome Server (TYGS) (https://tygs.dsmz.de/, accessed on 1 September 2023) [54]. Gene search and annotation were performed for all contigs longer than 500 bp using the Rapid Annotation using Subsystems Technology (RAST) server v.2.0 [56]. The SEED viewer was used for assignment of the predicted genes to functional categories [57]. Annular map of the circular chromosome of the *Sphingomonas* sp. AR_OL41 strain was constructed using the Proksee web service (https://proksee.ca/, accessed on 15 August 2023) [58]. Functional annotation was carried out using BlastKOALA (https://www.kegg.jp/blastkoala/, v. 3.0, 1 April 2023) [59].

The phylogenomic tree was constructed using an online service BV-BRC (PATRIC) v. 3.32.13a (https://www.bv-brc.org/, accessed on 1 August 2023) [60] via the Codon Tree method [61] with visualization by online service iTOL v. 6.8 (https://itol.embl.de/, accessed on 1 August 2023) [62]. The reconstruction of possible pathways of alkane metabolism was carried out based on a comparison of the genomes of the studied strains using NCBI online services (https://www.ncbi.nlm.nih.gov/genome/, release 258, 1 October 2023) [63], BV-BRC (PATRIC) 3.32.13a (https://www.bv-brc.org/, accessed on 1 August 2023), and KEGG (https://www.genome.jp/kegg/pathway.html, release 107.0, 1 July 2023) [64]. To visualize the structure of the *alkB* region of the genome, the Gene Graphics service was used [65].

### 2.7. Nucleotide Sequence Accession Numbers

The GenBank/EMBL/DDBJ accession number for the 16S rRNA gene sequence of the *Sphingomonas* sp. AR_OL41 strain was MZ411487. The GenBank/EMBL/DDBJ accession number of the genomic assembly of the *Sphingomonas* sp. AR_OL41 strain was GCF_029911635.1 (NZ_JARYTI010000000), and it was the first version described in this paper. 

## 3. Results and Discussion

Morphology, physiology, and growth below 0 °C of aerobic hydrocarbon-oxidizing bacteria *Pseudomonas frederiksbergensis* Ar-K7, *Rhodococcus yunnanensis* Ar-K9, *Arthrobacter alpinus* Ar-K10, and *Sphingomonas* sp. AR_OL41, previously isolated from samples of Arctic soils contaminated with diesel fuel [27], were investigated in more depth, and a genomic analysis was conducted of the *Sphingomonas* sp. AR_OL41 strain, which has the rare ability for members of this genus to use *n*-alkanes.

### 3.1. Characterization of the Strains

**Figure 1:**
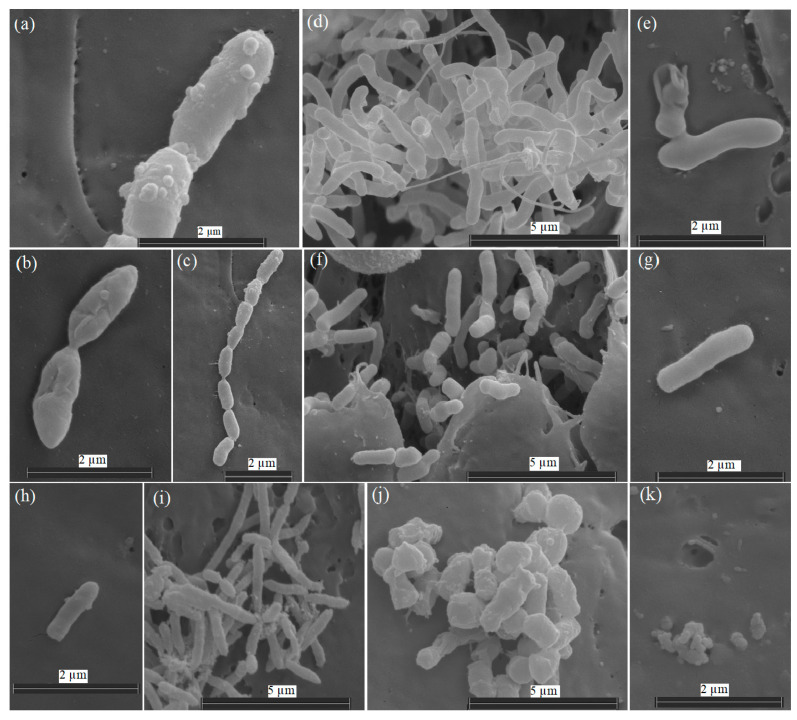
Scanning electron micrographs of cells of *Pseudomonas frederiksbergensis* Ar-K7 (**a**–**c**), *Rhodococcus yunnanensis* Ar-K9 (**d**–**g**), *Sphingomonas* sp. AR_OL41 (**h**,**i**), and *Arthrobacter alpinus* Ar-K10 (**j**,**k**), grown in a TEG medium at 25 °C (**b**,**d**,**e**,**i**,**j**) and −1.5 °C (**a**,**c**,**f**–**h**,**k**) for 14 days. The samples were examined under a scanning electron microscope (Quattro S, “Thermo Fisher Scientific”, Brno-Černovice, Czech Republic) at an accelerating voltage of 10 kV.

The studied strains Ar-K7, Ar-K9, Ar-10, and AR_OL41 were represented by non-spore-forming rods or cells with a rod–coccus cycle (Figure 1). The growth of strains at −1.5 °C was accompanied by changes in the number, morphology and structure of the cell surface compared with the growth at 25 °C. Visual observations with an electron microscope showed that the strains reacted differently to low temperatures. Thus, the cell length of the *P. frederiksbergensis* Ar-K7 strain was preserved at −1.5 °C (1.2–2.1 µm), but the surface became rougher, and the cell division process was disrupted, which led to the appearance of chains of up to 8 cells, uncharacteristic for the strain (Figure 1a–c). At 25 °C, the cells of the *R. yunnanensis* Ar-K9 strain formed microcolonies in the niches of Teflon cubes, and the cells were connected to each other by nanotubes. At a negative temperature, such cellular interaction did not occur, the maximum cell length decreased (from 1.0–3.4 µm to 1.0–2.7 µm), and the cell surface became rougher (Figure 1d–g).

In the *A*. *alpinus* Ar-K10 strain at −1.5 °C, the cell size also decreased from 0.7–1.5 µm (at 25 °C) to 0.2 µm (Figure 1j,k) Growth of the *Sphingomonas* sp. strain AR_OL41 at 25 °C was accompanied by the formation of colonies on the surface of the Teflon cubes, whereas the combination of two stress factors at once—low temperature and increased salinity (1% NaCl, *w*/*v*)—led to a decrease in the number of cells and their length (from 0.9–2.1 µm to 0.9–1.5 µm), and the ends of the cells became more rounded (Figure 1h,i). Visual observations showed a lower effect of negative temperature on the *R*. *yunnanensis* Ar-K9 strain, whose cell number remained high.

**Figure 2:**
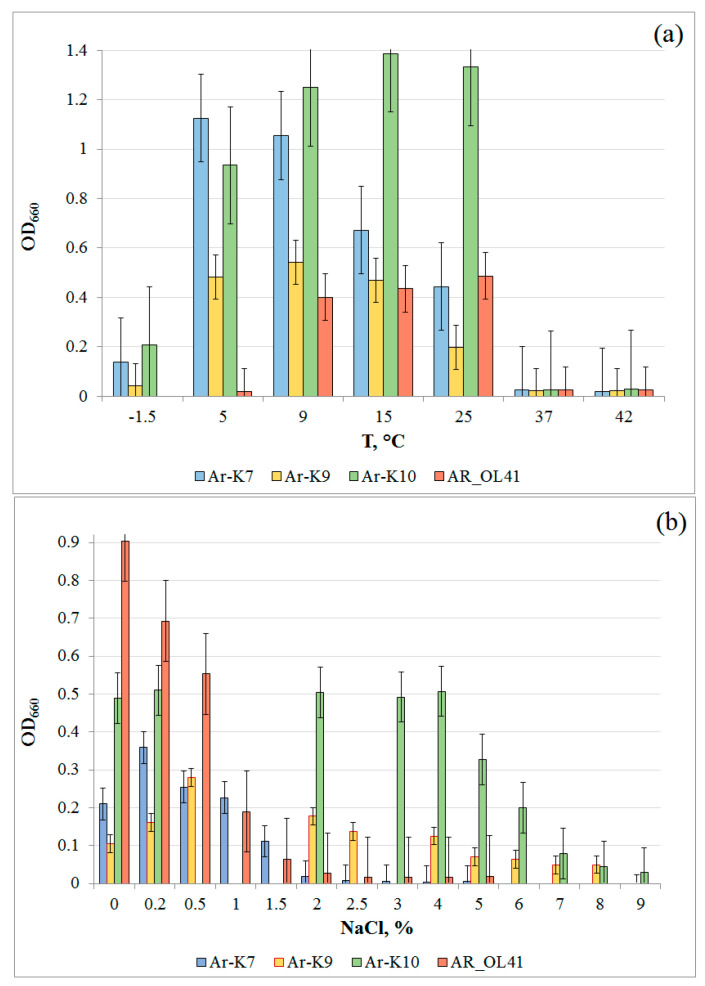
Growth of *Pseudomonas frederiksbergensis* Ar-K7, *Rhodococcus yunnanensis* Ar-K9, *Arthrobacter alpinus* Ar-K10, and *Sphingomonas* sp. AR_OL41 at various temperatures (**a**) and NaCl concentrations (*w*/*v*, %) (**b**).

The strains were psychrotolerant (Figure 2a) and grew at subzero temperatures but accumulated less biomass compared to optimal conditions. The temperatures optimal for the growth of Ar-K7, Ar-K9, and AR_OL41 strains were 5 °C, 5–15 °C, and 9–25 °C, respectively; the Ar-K10 strain grew optimally in a wide temperature range, from 5 to 25 °C. The temperature ranges for the growth of these strains were wider than those given for phylogenetically close type strains of *Pseudomonas frederiksbergensis* (4–30 °C), *Rhodococcus yunnanensis* (10–40 °C), *Arthrobacter alpinus* (1–25 °C), and *Sphingomonas alpina* (1–30 °C) [35,66,67,68]. The strains grew in the absence of NaCl in the medium (Figure 2b). *P*. *frederiksbergensis* Ar-K7 and *Sphingomonas* sp. AR_OL41 grew at low salt content in the medium not exceeding 1–1.5% NaCl (*w*/*v*), whereas the strains *R. yunnanensis* Ar-K9 and *A*. *alpinus* Ar-K10 grew in the presence of 4% and 6% NaCl, respectively.

The growth rates of Ar-K7 and Ar-K9 strains on the liquid TEG medium in periodic culture at 25 °C were higher than at 9 °C (see Figure S1). In the first 75 h of cultivation, the growth rates of the Ar-K7 strain at 9 and 25 °C were almost identical, whereas the Ar-K10 strain, in contrast, grew better at 9 °C. For all strains, the stationary phase occurred much later at a lower incubation temperature. The cell yields of Ar-K9, Ar-K10, and AR_OL41 strains at 9 °C were significantly higher than at 25 °C.

To understand the ecological role that isolated bacteria can play in the natural ecotope, their enzymatic activities—the ability to grow on natural biopolymers and participate in the transformations of phosphorus cycle compounds—were characterized.

The studied strains possessed a wide range of enzymes involved in the degradation of various proteins and carbohydrates (Table S1). In this study, the intraspecific variability of the enzymatic apparatus of the isolates from that of the type strains of the respective species was revealed. Thus, the Ar-K7 strain, unlike the *P*. *frederiksbergensis* JAJ28^T^ type strain [66], did not hydrolyze gelatin but contained arginine dehydrolase and did not form acid from glucose. The Ar-K9 strain showed β-glucosidase activity, unlike the *R. yunnanensis* YIM 70056^T^ strain [67]. The most complete set of enzymes was detected in the *A. alpinus* Ar-K10 strain, which—unlike the *A. alpinus* S6-3^T^ strain [68]—had lipase (C14), trypsin, and α-chymotrypsin activity. It also utilized citrate; however, it lacked urease. The AR_OL41 strain differed from the *Sphingomonas alpina* S8-3^T^ strain [35] by the presence of α-glucosidase and the absence of lipase (C14), trypsin, α-chymotrypsin, and gelatinase. The studied strains possessed alkaline and acid phosphatases, as well as arylamidases, which play an important role in the mineralization of phosphorus and nitrogen in soils. It was previously shown that microbial arylamidases can serve as an indicator of the degree of mineralization of organic nitrogen in soils [69]. It is known that alpha-galactosidases are responsible for the cleavage of glycolipids and polysaccharides and that beta-glucosidase cleaves cellulose; therefore, the use of natural biopolymers that can occur in the region (chitin, xylan, cellulose) as substrates has been tested. All studied strains grew on chitin; microcrystalline cellulose was used by three strains (except Ar-K9). Only the Ar-K7 and AR_OL41 strains grew on xylan (Table S1).

In polar regions, microorganisms face the need to protect themselves from reactive oxygen species because the solubility of gases in water increases with decreasing temperature [70]. The studied strains possessed the enzymes catalase and oxidase. An important ecological function of microorganisms in Arctic conditions may be the solubilization of inorganic phosphates (components of rocks), making them accessible to plants or other microorganisms and acting as a biofertilizer [71]. The ability of organotrophic bacteria to dissolve mineral phosphates is possible in the presence of carbon sources, which can be both natural polymers and alkanes of petroleum products. To test the phosphatase activity, calcium phosphate Ca_3_(PO_4_)_2_ was used, which is part of minerals such as phosphorite, apatite, and hydroxyapatite. The growth of the *P. frederiksbergensis* Ar-K7 and *A. alpinus* Ar-K10 strains was accompanied by the formation of an enlightenment zone in a solid NBRIP medium, which indicated the release of phosphorus ions. The Ar-K9 and AR_OL41 strains could not dissolve phosphates. Phosphatase activity has previously been shown for psychrophilic bacteria of the genera *Acinetobacter*, *Bacillus*, *Pantoea*, *Pseudomonas*, *Rahnella*, and *Serratia* [72].

### 3.2. Crude Oil Degradation

The use of bioremediation methods from hydrocarbon pollution in the Arctic conditions faces a number of difficulties. The vegetation season of the Arctic region is very short. The effective microbial use of hydrocarbons occurs here for several months. Therefore, methods of stimulating the native microflora (especially in the Antarctic region, where the use of non-indigenous microorganisms is prohibited [73]) or cultivation of native psychrophilic hydrocarbon-oxidizing microorganisms originally adapted to the conditions of the region and returning them to contaminated soil gain an advantage.

In this work, the oxidation of crude oil by Arctic strains at temperatures of −1.5 °C and 9 °C was investigated. The process of oil oxidation at −1.5 °C was less efficient than at 9 °C (Figure S2a–c). The Ar-K7 strain utilized *n*-alkanes with a chain length of C_15_–C_22_, using no more than 20% of the initial number of individual *n*-alkanes (Figure S2a). The Ar-K9 strain preferably used C_11_–C_13_ *n*-alkanes, and the Ar-K10 strain used C_16_–C_30_ *n*-alkanes (Figure S2b,c). The general trend was a narrowing of the spectrum of alkanes used at negative temperatures and a decrease in hydrocarbon consumption. At 9 °C, the strains degraded more unbranched and branched C_11_–C_30_ *n*-alkanes than at −1.5 °C. The formation of biofilms and biosurfactants [19] by the studied strains indicates their possible participation in soil self-purification and use in biotechnologies for the bioremediation of oil-polluted low-temperature habitats.

Because the negative temperature combined with high salinity led to a strong suppression of the growth of the *Sphingomonas* sp. AR-OL41 strain, we analyzed its ability to degrade oil at 9 and 25 °C. As can be seen from Figure S2d, the AR-OL41 strain used only medium-chain C_11_–C_13_ *n*-alkanes, whereas biodegradation at 9 °C was slightly higher than at 25 °C, which correlates with the results of determining the optimal growth temperature in periodic culture, which showed a higher cell yield at 9 °C than at 25 °C. The degradation of aromatic compounds was not estimated in this study. However, previous studies have confirmed the degradation of various aromatic compounds by *Sphingomonas* spp. strains [37,74,75].

To assess the ability of three strains to use hydrocarbons in conditions close to natural ones, a model experiment was conducted to clean quartz sand from oil pollution at 9 °C (Table S2). The initial number of *P. frederiksbergensis* Ar-K7 and *A. alpinus* Ar-K10 strains was 10^7^–10^8^ cells/g of sand. Both strains survived at low temperatures, even in the absence of a substrate: a high number remained throughout the experiment, including in the control without oil. Perhaps the cells used dead biomass while maintaining viability. The initial number of *R. yunnanensis* Ar-K9 strain was 10^3^ cells/g of sand, but after 30 days, it increased to 10^7^ cells/g of sand. At the same time, in the control without oil, it decreased to zero. After 30 days of incubation in oil, up to 82 mg of acetate and about 10 mg of ethanol were registered per g of sand. After 60 days, these values decreased. Apparently, the resulting oil oxidation products were also used as a substrate.

The maximum formation of biofilms by Ar-K7 and Ar-K10 strains was registered after 60 days, and for the Ar-K9 strain, after 30 days of incubation; after 60 days, the indicator value also decreased. The analysis of the aliphatic fraction of oil showed a decrease in the content of *n*-alkanes over time (Figure S3). The maximum decrease in *n*-alkanes was demonstrated through the growth of the *R. yunnanensis* Ar-K9 strain. The maximum loss of oil (about 80 mg/100 g of sand) as a result of 60 days of cultivation was noted by the weight method for the *P. frederiksbergensis* Ar-K7 strain. In addition to the aliphatic fraction, the Ar-K7 strain probably also used aromatic hydrocarbons. The growth was accompanied by the formation of biofilms on the sand (Table S2, Figure S4).

### 3.3. Genome Statistics and Functional Characterization of the Sphingomonas sp. AR_OL41

**Figure 3:**
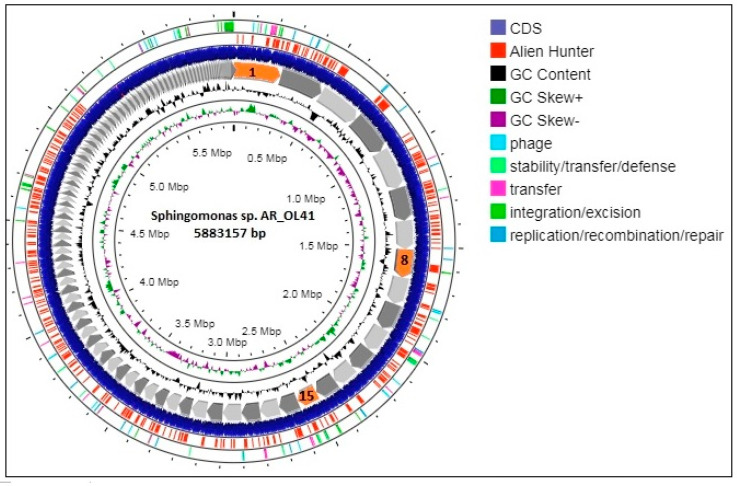
Graphic map of the circular chromosome of the *Sphingomonas* sp. AR_OL41 strain. The contigs JARYTI010000001, JARYTI010000008, and JARYTI010000015, on which the degradation genes of *n*-alkanes (1) and phenylpropanoids (8 and 15) are localized, are highlighted in red.

The genome of the AR_OL41 strain consisted of 188 contigs with a total size of 5,883,157 bp and had an average coverage of 52×, an assembly completeness of 98.8%, a contamination of 4.16%, and a G + C content of 65.5%. The genome contained 5743 genes, including 5613 protein-coding genes, 3 rRNAs (5S, 16S, and 23S), 52 tRNAs, 3 noncoding RNAs, 58 noncoding genes, and 127 pseudogenes. On the map of the circular chromosome of the AR_OL41 strain (Figure 3) obtained using the Proksee server, according to the results of the built-in Alien Hunter module, a significant number of sections of foreign DNA were revealed—presumably obtained through horizontal gene transfer—as were genes presumably contributing to this transfer.

**Figure 4:**
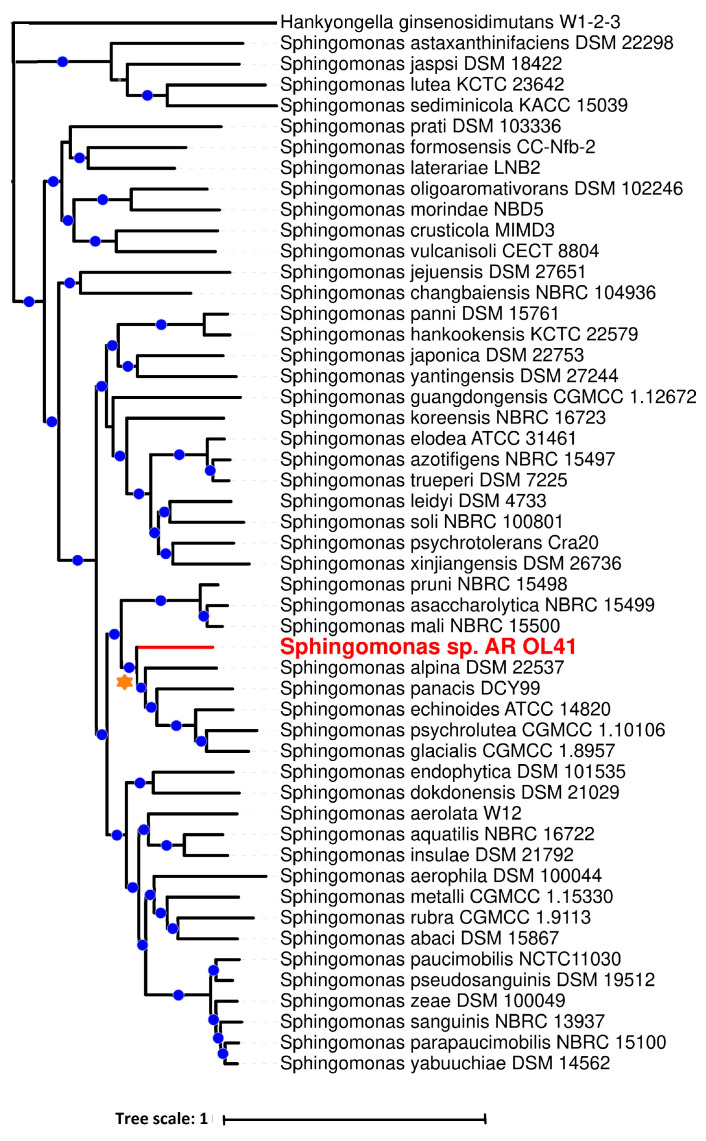
A phylogenomic tree of representative type strains of the species of the *Sphingomonas* genus, showing the phylogenetic position of the *Sphingomonas* sp. AR_OL41 strain (marked in red), constructed using internet services BV-BRC (PATRIC) version 3.32.13a and iTOL version 6.8. The cluster of species closest to the AR_OL41 strain is marked with an orange asterisk. The blue dots indicate branching, confirmed by bootstrap values >85. Bar indicates the number of nucleotide substitutions per site. The tree was rooted using the *Henkyongella ginsenosidimutans* W1-2-3 genome as the outgroup.

When using the TYGS server, the AR_OL41 genome was the closest to the genome of the *Sphingomonas alpina* S6-3^T^ strain. The dDDH value between strain AR_OL41 and the *S. alpina* S6-3^T^ strain was 20.9%, and the ANI value was 81.9%. These values are lower than the proposed and generally accepted species boundaries for dDDH and ANI [76] and indicate the possibility of isolating the AR_OL41 strain into a new species of the genus *Sphingomonas*. On the phylogenetic tree of type strains of validly published species of the genus *Sphingomonas* (Figure 4), the AR_OL41 strain formed a separate branch within a *S. alpina*, *S. panacis*, *S. echinoides*, *S. glacialis*, and *S. psychrolutea* cluster. The ranges of ANI values for the members of this cluster were 74.7–81.9%, and dDDH was 20.2–21.2%, which also confirms the assumption that the *Sphingomonas* sp. AR_OL41 strain could be isolated into a new species.

Functional annotation of the *Sphingomonas* sp. AR_OL41 genome, performed with RASTtk, identified five main subsystems, including 257 genes involved in the carbohydrate metabolism, 240 genes of the metabolism of amino acids and their derivatives, 199 genes of membrane transport, 195 genes associated with protein metabolism, and 172 genes of the metabolism of cofactors, vitamins, prosthetic groups, and pigments (Figure S5). In addition, this study found 100 genes of respiration; 93 genes of stress response; 89 genes associated with fatty acids, lipids, and isoprenoids metabolism; and 333 genes related to various functional subsystems.

An analysis of the enzyme composition of metabolic pathways based on the results of functional prediction of proteins using BlastKOALA suggests the genome of the AR_OL41 strain carries complete sets of genes responsible for complete pathways of carbohydrate metabolism: glycolysis (Embden–Meyerhoff pathway), pyruvate oxidation, the tricarboxylic acid cycle, the pentose phosphate pathway, the Entner–Doudoroff pathway, and the glyoxylate cycle. In addition, enzymes that catabolize cellulose, cellodextrin, and cellobiose are annotated in this subsystem. The subsystem “Amino acids and their derivatives” annotates the key genes responsible for the complete pathways of biosynthesis of basic amino acids. Membrane transport enzymes are annotated for phosphates, lipoproteins, lipopolysaccharides, phospholipids, ribose, and D-xylose. The genes of the complete biosynthesis pathways of NAD, biotin, tetrahydrofolate, and molybdenum cofactor have also been annotated. In the subsystem “Fatty acids, lipids, and isoprenoids metabolism”, enzymes of both the complete biosynthesis pathways of fatty acids and phosphatidylethanolamine and the complete pathways of beta-oxidation of fatty acids, as well as degradation of *n*-alkanes, are annotated. In the “Nitrogen metabolism” subsystem, only the enzymes of ammonium to glutamate oxidation are annotated, and the genes of the enzymes responsible for nitrogen fixation—as well as nitrate- and nitrite-reduction—have not been identified.

In the “Sulfur metabolisms” subsystem, there are annotated assimilatory sulfate reduction enzymes, including adenylyl sulfate transferase (EC: 2.7.7.4), a key sulfate reduction enzyme that catalyzes the conversion of sulfate to adenylyl sulfate, as well as adenosyl sulfate kinase (EC: 2.7.1.25) and phosphoadenosine phosphosulfate reductase (EC: 1.8.4.8), reducing sulfite to sulfide. In addition, enzymes that catabolize alkanesulfonate, thiosulfate, and dimethylsulfone to sulfite have been annotated.

In the “Degradation of aromatic compounds” subsystem, the complete pathways are annotated only for the degradation of trans-cinnamic and 3-phenylpropanoic acids. These acids are part of the phenylpropanoid family, which unites a wide range of C_6_–C_3_ aromatic compounds synthesized by plants from the amino acid phenylalanine and serve as an integral element of various natural polymers, in particular lignin. Bacterial catabolism of these compounds plays an important role in the carbon cycle by which both natural aromatics and many industrial pollutants are degraded [77]. The complete set of enzymes of these pathways is specific to the AR_OL41 strain because the enzyme 3-carboxyethylcatechol 2,3-dioxygenase (EC: 1.13.11.16) is absent in the genomes of other reference type strains of the genus *Sphingomonas* (Figure S6).

### 3.4. alkB Genes in Genomes of Sphingomonadaceae Bacteria

Many members of the *Sphingomonadaceae* family have been isolated from soils contaminated with oil and petroleum products as degraders of aromatic compounds, but their ability to use *n*-alkanes has been significantly less studied [78]. At the time of this study, the PATRIC database contained information on 2565 genomes of bacteria of the *Sphingomonadaceae* family, including 2375 genomes of cultured and uncultivated representatives belonging to 21 genera, as well as 190 unclassified strains. *alkB* genes determining the most common alkane-1 monooxygenase were annotated in a total of 44 genomes of bacteria of the genera *Blastomonas*, *Novosphingobium*, *Parasphingopyxis*, *Parasphingorhabdus*, *Sphingobium*, *Sphingomicrobium*, *Sphingomonas*, *Sphingopyxis*, and *Sphingorhabdus*, including the genome of the studied strain *Sphingomonas* sp. AR_OL41. Of the 1171 bacterial genomes of the most numerous genus *Sphingomonas*, only 6 had *alkB* genes encoding alkane 1-monooxygenase annotated. The presence of *alkB* genes is not a characteristic feature for most genera of the *Sphingomonadaceae* family, including the *Sphingomonas* genus; therefore, the ability to degrade *n*-alkanes is a rare feature of the metabolism of individual bacterial strains of this family.

According to the KEGG database, the degradation of *n*-alkanes is included in the metabolic pathway “Degradation of fatty acids” (Figure S7). The enzyme alkane monooxygenase (EC: 1.14.15.3) participates in this process both at the initial stage of alkane oxidation and at the terminal stage of hydroxylation of fatty acids into omega-hydroxy fatty acids. The participation of this enzyme in the metabolism of fatty acids of the *Sphingomonas* sp. AR_OL41 strain is assumed based on the annotation of the *alkB* gene in its genome, which determines alkane-1 monooxygenase. Together with alkane-1 monooxygenase, the protein rubredoxin and the enzyme ferredoxin reductase (EC: 1.18.1.3)—as well as other enzymes of alkane metabolism, alcohol dehydrogenase (EC: 1.1.1.1), and aldehyde dehydrogenase (EC: 1.2.1.3)—are assumed to be part of the hydrolase system of alkane oxidation of the AR_OL41 strain. In addition, it is assumed that at the terminal stage of the *n*-alkane degradation process, the fatty acid is hydroxylated to alpha-hydroxy fatty acid using another cytochrome P450/NADPH-cytochrome P450 reductase, unspecific P450 monooxygenase (EC: 1.14.14.1).

**Figure 5:**
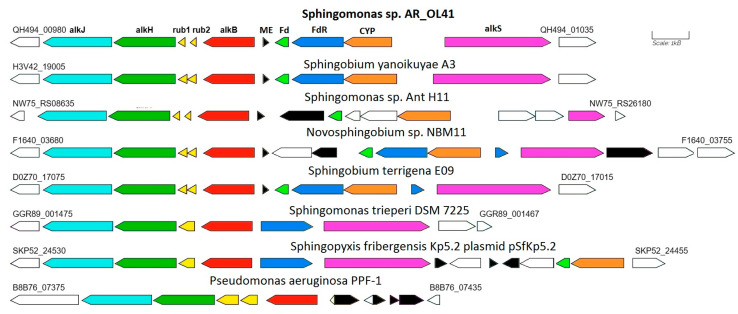
Comparison of the structure of genome regions containing annotated alkane-1 monoxygenase genes in bacterial strains of the *Sphingomonadaceae* family. The genes of enzymes presumably involved in the oxidation of *n*-alkanes are highlighted in color. Designations: *alkB*—alkane-1 monooxygenase; *rub*—rubredoxin; *alkH*—aldehyde dehydrogenase; *alkJ*—alcohol dehydrogenase; Fd—ferredoxin; FdR—ferredoxin reductase; CYP—presumably cytochrome P450; *alkS*—transcriptional regulator; ME–proteins of mobile elements.

In the genome of the *Sphingomonas* sp. AR_OL41 strain, one alkane-1-monooxygenase (*alkB*) gene with a length of 1212 bp was annotated. In the GenBank database, the translated amino acid sequence of this gene (403 a.a.) was close to similar sequences of different strains of the genera *Sphingobium* and *Novosphingobium* (98.8–99.3% identity). In the genome of the *Sphingomonas* sp. AR_OL41 strain, the *alkB* alkane hydroxylase gene cluster is localized on the JARYTI010000001 contig. In addition to the *alkB* gene encoding alkane-1 monooxygenase, this cluster includes two rubredoxin genes (*rub1* and *rub2*) as well as genes for further catabolism of *n*-alkanes—aldehyde dehydrogenase (*alkH*) and alcohol dehydrogenase (*alkJ*) (see Figure 5).

The alkane hydroxylase gene cluster is flanked by the OmpW family outer membrane protein gene. In contrast, the cluster of genes encoding ferredoxin, ferredoxin reductase, and putative cytochrome P450, as well as the oppositely directed transcriptional regulator *alkS* gene, are adjacent to this cluster and are separated from it by the gene of the mobile element protein (transposases of the IS5/IS1182 family). This cluster is flanked by the alpha/beta hydrolase gene and a group of mobile elements (transposases).

Some strains of the genera *Sphingobium* and *Novosphingobium* had similar structures of *alkB* regions, the translated sequences of which were almost identical to those of the *Sphingomonas* sp. AR_OL41 strain. In the genome of *Sphingomonas trueperi* DSM 7225, the *alkB* region was partially similar to that of the *Sphingomonas* sp. AR_OL41 strain; however, no cluster of cytochrome P450 genes was annotated in it.

The key *n*-alkane degradation gene, *alkB*, and its accompanying genes are localized on the JARTYI010000001 contig (region QH494-00980–QH494-01035). According to the results of the analysis using the Prokka service with the Alien Hunter module, the entire AlkB region is localized in a section of the genome that is presumably foreign DNA (Figure S8). This site is flanked by genes of proteins of mobile elements (transposases), which confirms the possibility of its transfer from other genomes. In addition, another transposase gene separates the AlkB cluster from the CYP cluster, so it can be assumed the transfer process was repeated. Plasmids carrying alkane hydroxylase genes could be involved in the transfer process. This assumption is supported, in particular, by data on the genome structure of the *Sphingopyxis fribergensis* Kp5.2^T^ type strain [79], which contains a chromosome and a plasmid. At the same time, the alkane monooxygenase genes on the chromosome are not annotated, whereas the AlkB cluster is annotated on the plasmid identically to that from the chromosome of *Sphingomonas trueperi* DSM 7225 and, accordingly, partially similar to that from the genome of *Sphingomonas* sp. AR_OL41. At the same time, the AlkB cluster from the plasmid of the *Sphingopyxis fribergensis* Kp5.2 strain was flanked by genes of mobile elements that could facilitate its transfer to the genomes of other strains of the *Sphingomonadaceae* family. The transfer donor could be bacteria characterized by the oxidation of *n*-alkanes—in particular, *Pseudomonas* spp. or *Rhodococcus* spp. alkane monooxygenase genotypes—which are prevalent in Arctic soils [80].

### 3.5. Cold Stress Protection Genes in the Genome of Sphingomonas sp. AR_OL41

For the *Sphingomonas* sp. AR_OL41 strain, the adaptation to cold conditions was also analyzed at the genome level. The results of the search for genes responsible for protection against cold shock [81] are shown in Table S3. Genes for DNA transcription and replication regulator (*dna*), recombination factor A (*recA*), topoisomerases (*gyrA*), and genes encoding the DNA-binding protein HU-beta were found. In addition, several copies presented genes of the CspA family, cold shock proteins that act as chaperones of nucleic acids to prevent the formation of secondary mRNA structures at low temperatures and contribute to the initiation of translation. Other genes encoding molecular chaperones in psychrophilic conditions include *dnaK*, *dnaJ*, and peptidyl prolyl cis-trans, responsible for protein folding. The genes determining the synthesis of transcription initiation factor I (*infA*) and ribosome-binding factor A (*rbfA*), protein biosynthesis, desaturation of membrane lipids, formation of exopolysaccharides, and pyruvate metabolism enzymes (*pdhA*, *pdhB*, and *pdhC*) were also annotated, and their participation in protection against cold stress was also shown. Thus, in the genome of the Arctic strain *Sphingomonas* sp. AR_OL41, there is a wide range of genes in different functional categories that contribute to the adaptation of bacteria to low-temperature conditions.

## 4. Conclusions

In this work, the physiological characteristics and growth at low temperatures of hydrocarbon-oxidizing bacteria *Pseudomonas frederiksbergensis* Ar-K7, *Rhodococcus yunnanensis* Ar-K9, *Arthrobacter alpinus* Ar-K10, and *Sphingomonas* sp. AR_OL41 isolated from oil-contaminated polar soils were determined. It is shown that the strains are adapted to the conditions of the Arctic region. They could grow at low temperatures (up to −1.5 °C) and high NaCl contents in the medium, use biopolymers (xylan, chitin, cellulose) and crude oil, and dissolve phosphate minerals. When growing in oil, the strains formed biosurfactants and biofilms. Lowering the incubation temperature led to a narrowing of the spectrum of oil *n*-alkanes used. The ability to use *n*-alkanes is rare for members of the genus *Sphingomonas*. Experimental observations of *n*-alkanes utilization by *Sphingomonas* sp. AR_OL41 strain were confirmed by the genomic sequencing using the Illumina HiSeq 2500 platform and bioinformatics analysis. Among 2565 genomes of bacteria of the *Sphingomonadaceae* family, the presence of *alkB* genes determining the most common alkane-1 monooxygenase was revealed in 44 genomes only. Focusing on the ecological functions of the AR_OL41 strain, a complete cluster of *alkB* genes was found in the genome, as were genes that determine cold shock proteins, desaturation of membrane lipids, synthesis of exopolysaccharides, and pyruvate metabolism. The ANI and dDDH values of strain AR_OL41 in comparison to other members of *Sphingomonas* spp. were lower than the established cutoff values for prokaryotic species delineation, indicating that the AR_OL41 strain represents a novel species of the genus *Sphingomonas*. Genomic information obtained in this study could be used to determine the taxonomic position of *Sphingomonas* sp. AR_OL41 and to obtain a deeper understanding of its metabolic potential and ability to contribute to the self-cleaning of hydrocarbon-contaminated Arctic soils. This study highlights the biotechnological potential of *Pseudomonas frederiksbergensis* Ar-K7 and *Arthrobacter alpinus* Ar-K10, which grow efficiently in crude oil at low temperatures, for application in bioremediation of the northern territories.

## Data Availability

The whole-genome shotgun project of strain AR_OL41 has been deposited at DDBJ/EMBL/GenBank under the accession GCA_029911635.1, and it is the first version described in this paper.

## Supplementary Materials

The following supporting information can be downloaded at: https://www.mdpi.com/article/10.3390/microorganisms12010079/s1, Figure S1: Growth curves of the strains *Pseudomonas frederiksbergensis* Ar-K7 (a), *Rhodococcus yunnanensis* Ar-K9 (b), *Arthrobacter alpinus* Ar-K10 (c), and *Sphingomonas* sp. AR_OL41 (d) in the TEG liquid medium at 9 °C and 25 °C; Figure S2: Residual content of *n*-alkanes in crude oil biodegraded by *Pseudomonas frederiksbergensis* Ar-K7 (a), *Rhodococcus yunnanensis* Ar-K9 (b), *Arthrobacter alpinus* Ar-K10 (c), and *Sphingomonas* sp. AR_OL41 (d) at −1.5 °C, 9 °C, and 25 °C; Figure S3: Residual content of *n*-alkanes (%) in crude oil biodegraded by *Pseudomonas frederiksbergensis* Ar-K7 (a), *Rhodococcus yunnanensis* Ar-K9 (b), and *Arthrobacter alpinus* Ar-K10 (c) during remediation of polluted sand; Figure S4: Scanning electron micrographs of biofilms of the strains *Pseudomonas frederiksbergensis* Ar-K7 (a) and *Rhodococcus yunnanensis* Ar-K9 (b) after 30 days of cultivation in oil-contaminated sand. The samples were examined under a scanning electron microscope (Quattro S, “Thermo Fisher Scientific”, Brno-Černovice, Czech Republic) with accelerating voltage 15 kV; Figure S5: Subsystems of *Sphingomonas* sp. AR_OL41 based on RAST database; Figure S6: KEGG-map of phenylalanine metabolism (id 00360) with degradation modules of trans-cinnamic and phenylpropionic acids based on genome analysis of the strain *Sphingomonas* sp. AR_OL41; Figure S7: KEGG-map of degradation of fatty acids and *n*-alkanes (id 00071) based on genome analysis of the strain *Sphingomonas* sp. AR_OL41; Figure S8: Localization of *n*-alkane degradation genes on the graphic map of the circular chromosome of the strain *Sphingomonas* sp. AR_OL41. The genes of mobile elements (ME) are highlighted in green. Designations: *alkB*—alkane-1 monooxygenase; *rub*–rubredoxin; *alkH*—aldehyde dehydrogenase; *alkJ*—alcohol dehydrogenase; Fd—ferredoxin; FdR—ferredoxin reductase; CYP–presumably cytochrome P450; *alkS*—transcriptional regulator; ME—proteins of mobile elements; Table S1: Phenotypic characteristics of hydrocarbon-oxidizing bacteria *Pseudomonas frederiksbergensis* Ar-K7, *Rhodococcus yunnanensis* Ar-K9, *Arthrobacter alpinus* Ar-K10, and *Sphingomonas* sp. AR_OL41; Table S2: Physiological growth parameters of *Pseudomonas frederiksbergensis* Ar-K7, *Rhodococcus yunnanensis* Ar-K9, and *Arthrobacter alpinus* Ar-K10 in oil-contaminated sand; Table S3: Genes implicated in the adaptation to cold environments in the genome of *Sphingomonas* sp. AR_OL41.

## References

[1] Henderson J., Loe J. (2014). The prospects and challenges for arctic oil development. Oxf. Inst. Energy Stud..

[2] Bashkin V.N., Galiulin R.V. (2021). Innovative geoecological risk assessment in technogenesis for green economy progress. Green Econ. Era Fourth Ind. Revolut..

[3] Mohn W.W., Stewart G.R. (2000). Limiting factors for hydrocarbon biodegradation at low temperature in Arctic soils. Soil Biol. Biochem..

[4] Naseri M., Barabadi A., Barabady J. (2014). Bioremediation treatment of hydrocarbon-contaminated Arctic soils: Influencing parameters. Environ. Sci. Pollut. Res. Int..

[5] Vasilyeva G., Kondrashina V., Strijakova E., Ortega-Calvo J.J. (2020). Adsorptive bioremediation of soil highly contaminated with crude oil. Sci. Total Environ..

[6] Ławniczak Ł., Wózniak-Karczewska M., Loibner A.P., Heipieper H.J., Chrzanowski Ł. (2020). Microbial degradation of hydrocarbons—Basic principles for bioremediation: A Review. Molecules.

[7] Van Stempvoort D., Biggar K. (2008). Potential for bioremediation of petroleum hydrocarbons in groundwater under cold climate conditions: A review. Cold Reg. Sci. Technol..

[8] Greer C.W., Whyte L.G., Niederberger T.D., Timmis K.N. (2010). Microbial communities in hydrocarbon-contaminated temperate, tropical, alpine, and polar soils. Handbook of Hydrocarbon and Lipid Microbiology.

[9] Bell T.H., Yergeau E., Maynard C., Juck D., Whyte L.G., Greer C.W. (2013). Predictable bacterial composition and hydrocarbon degradation in Arctic soils following diesel and nutrient disturbance. ISME J..

[10] Mohammed D., Hicham E.A., Naima E.G., Encarnação T., Canelas Pais A. (2023). Biodegradation of environmental pollutants by marine yeasts. Marine Organisms: A Solution to Environmental Pollution? Environmental Challenges and Solutions.

[11] Myazin V.A., Korneykova M.V., Chaporgina A.A., Fokina N.V., Vasilyeva G.K. (2021). The Effectiveness of biostimulation, bioaugmentation and sorption-biological treatment of soil contaminated with petroleum products in the Russian Subarctic. Microorganisms.

[12] Namsaraev Z., Bobrik A., Kozlova A., Krylova A., Rudenko A., Mitina A., Saburov A., Patrushev M., Karnachuk O., Toshchakov S. (2023). Carbon emission and biodiversity of Arctic soil microbial communities of the Novaya Zemlya and Franz Josef Land Archipelagos. Microorganisms.

[13] Semenova E.M., Babich T.L., Sokolova D.S., Ershov A.P., Raievska Y.I., Bidzhieva S.K., Stepanov A.L., Korneykova M.V., Myazin V.A., Nazina T.N. (2022). Microbial communities of seawater and coastal soil of Russian Arctic region and their potential for bioremediation from hydrocarbon pollutants. Microorganisms.

[14] Korneykova M.V., Myazin V.A., Fokina N.V., Chaporgina A.A., Nikitin D.A., Dolgikh A.V. (2023). Structure of microbial communities and biological activity in tundra soils of the Euro-Arctic region (Rybachy peninsula, Russia). Microorganisms.

[15] Rike A.G., Haugen K.B., Børresen M., Engene B., Kolstad P. (2003). In situ biodegradation of petroleum hydrocarbons in frozen arctic soils. Cold Reg. Sci. Technol..

[16] Rike A.G., Haugen K.B., Engene B. (2005). In situ biodegradation of hydrocarbons in arctic soil at sub-zero temperatures–field monitoring and theoretical simulation of the microbial activation temperature at a Spitsbergen contaminated site. Cold Reg. Sci. Technol..

[17] Rivkina E.M., Friedmann E.I., McKay C.P., Gilichinsky D.A. (2000). Metabolic activity of permafrost bacteria below the freezing point. Appl. Environ. Microbiol..

[18] Brakstad O.G., Bonaunet K. (2006). Biodegradation of petroleum hydrocarbons in seawater at low temperatures (0–5 °C) and bacterial communities associated with degradation. Biodegradation.

[19] McFarlin K.M., Prince R.C., Perkins R., Leigh M.B. (2014). Biodegradation of dispersed oil in Arctic seawater at −1°C. PLoS ONE.

[20] Jesus H.E., Carreira R.S., Paiva S.S.M., Massone C., Enrich-Prast A., Peixoto R.S., Rodrigues J.L.M., Lee C.K., Cary C., Rosado A.S. (2021). Microbial succession under freeze–thaw events and its potential for hydrocarbon degradation in nutrient-amended Antarctic soil. Microorganisms.

[21] Gerdes B., Brinkmeyer R., Dieckmann G., Helmke E. (2005). Influence of crude oil on changes of bacterial communities in Arctic sea-ice. FEMS Microbiol. Ecol..

[22] Colla G., Rouphael Y., Canaguier R., Svecova E., Cardarelli M. (2014). Biostimulant action of a plant-derived protein hydrolysate produced through enzymatic hydrolysis. Front. Plant Sci..

[23] Colla T.S., Andreazza R., Bücker F., de Souza M.M., Tramontini L., Prado G.R., Frazzon A.P.G., Camargo F.A., Bento F.M. (2014). Bioremediation assessment of diesel–biodiesel-contaminated soil using an alternative bioaugmentation strategy. Environ. Sci. Pollut. Res..

[24] Myazin V.A., Isakova E.A., Vasilyeva G.K. (2020). Influence of granular activated carbon on the rate of bioremediation of soils in the Murmansk region, historically contaminated with oil products. Probl. Reg. Ekol..

[25] Myazin V.A., Ivanova L.A., Chaporgina A.A., Fokina N.V., Korneikova M.V., Evdokimova G.A. (2023). It’s Time to Heal the Arctic. Biological Methods of Cleaning and Remediation of Oil-Contaminated Areas. FRS KSC RAS, Apatity. https://rio.ksc.ru/data/documents/2_2022_13/%D0%BF%D0%BE%D1%80%D0%B0%20%D0%BE%D0%B7%D0%B4%D0%BE%D1%80%D0%B0%D0%B2%D0%BB%D0%B8%D0%B2%D0%B0%D1%82%D1%8C%20%D0%B0%D1%80%D0%BA%D1%82%D0%B8%D0%BA%D1%83_2023-%D0%905.pdf.

[26] Evdokimova G.A., Gershenkop A.S., Fokina N.V. (2012). The impact of bacteria of circulating water on apatite-nepheline ore flotation. J. Environ. Sci. Health A Tox. Hazard. Subst. Environ. Eng..

[27] Semenova E.M., Babich T.L., Sokolova D.S., Dobriansky A.S., Korzun A.V., Kryukov D.R. (2021). Microbial diversity of hydrocarbon-contaminated soils of the Franz Josef land Archipelago. Microbiology.

[28] Newsham K.K., Danielsen B.K., Biersma E.M., Elberling B., Hillyard G., Kumari P., Priemé A., Woo C., Yamamoto N. (2022). Rapid response to experimental warming of a microbial community inhabiting high Arctic patterned ground soil. Biology.

[29] Peeb A., Dang N.P., Truu M., Nõlvak H., Petrich C., Truu J. (2022). Assessment of hydrocarbon degradation potential in microbial communities in Arctic Sea ice. Microorganisms.

[30] Whyte L.G., Bourbonnie`re L., Greer C.W. (1997). Biodegradation of petroleum hydrocarbons by psychrotrophic *Pseudomonas* strains possessing both alkane (*alk*) and naphthalene (*nah*) catabolic pathways. Appl. Environ. Microbiol..

[31] Whyte L.G., Slagman S.J., Pietrantonio F., Bourbonnière L., Koval S.F., Lawrence J.R., Inniss W.E., Greer C.W. (1999). Physiological adaptations involved in alkane assimilation at a low temperature by *Rhodococcus* sp. strain Q15. Appl. Environ. Microbiol..

[32] Xu X., Liu W., Tian S., Wang W., Qi Q., Jiang P., Gao X., Li F., Li H., Yu H. (2018). Petroleum hydrocarbon-degrading bacteria for the remediation of oil pollution under aerobic conditions: A perspective analysis. Front. Microbiol..

[33] Mohapatra B., Phale P.S. (2021). Microbial degradation of naphthalene and substituted naphthalenes: Metabolic diversity and genomic insight for bioremediation. Front. Bioeng. Biotechnol..

[34] Pandolfo E., Caracciolo A.B., Rolando L. (2023). Recent advances in bacterial degradation of hydrocarbons. Water.

[35] Margesin R., Zhang D., Busse H. (2012). *Sphingomonas alpina* sp. nov., a psychrophilic bacterium isolated from alpine soil. Int. J. Syst. Evol. Microbiol..

[36] Zhou L., Li H., Zhang Y., Han S., Xu H. (2016). *Sphingomonas* from petroleum-contaminated soils in Shenfu, China and their PAHs degradation abilities. Environ. Microbiol..

[37] Asaf S., Numan M., Khan A.L., Al-Harrasi A. (2020). *Sphingomonas*: From diversity and genomics to functional role in environmental remediation and plant growth. Crit. Rev. Biotechnol..

[38] Somee M.R., Amoozegar M.A., Dastgheib S.M.M., Shavandi M., Maman L.G., Bertilsson S., Mehrshad M. (2022). Genome-resolved analyses show an extensive diversification in key aerobic hydrocarbon-degrading enzymes across bacteria and archaea. BMC Genom..

[39] Pfennig N., Lippert K.D. (1966). Über das Vitamin B12-Bedürfnis phototropher Schwefelbakterien. Arch. Mikrobiol..

[40] Sokolova D.S., Semenova E.M., Grouzdev D.S., Bidzhieva S.K., Babich T.L., Loiko N.G., Ershov A.P., Kadnikov V.V., Beletsky A.V., Mardanov A.V. (2021). Sulfidogenic microbial communities of the Uzen high-temperature oil field in Kazakhstan. Microorganisms.

[41] Appelbaum P.C., Stavitz J., Bentz M.S., von Kuster L.C. (1980). Comparison of four methods for identification of Gram-negative non-fermenters: Organisms less commonly encountered in clinical specimens. J. Clin. Microbiol..

[42] Nautiyal C.S. (1999). An efficient microbiological growth medium for screening phosphate solubilizing microorganisms. FEMS Microbiol. Lett..

[43] Semenova E.M., Ershov A.P., Sokolova D.S., Tourova T.P., Nazina T.N. (2020). Diversity and biotechnological potential of nitrate-reducing bacteria from heavy-oil reservoirs (Russia). Microbiology.

[44] Manucharova N.A., Bolshakova M.A., Babich T.L., Tourova T.P., Semenova E.M., Yanovich A.S., Poltaraus A.B., Stepanov A.L., Nazina T.N. (2021). Microbial degraders of petroleum and polycyclic aromatic hydrocarbons from sod-podzolic soil. Microbiology.

[45] Wang H., Cheng H., Wang F., Wei D., Wang X. (2010). An improved 3-(4,5-dimethylthiazol-2-yl)-2,5-diphenyl tetrazolium bromide (MTT) reduction assay for evaluating the viability of *Escherichia coli* cells. J. Microbiol. Meth..

[46] Plakunov V.K., Mart’yanov S.V., Teteneva N.A., Zhurina M.V. (2016). A universal method for quantitative characterization of growth and metabolic activity of microbial biofilms in static models. Microbiology.

[47] Lane D.J., Stackebrandt E., Goodfellow M. (1991). 16S/23S rRNA sequencing. Nucleic Acid Techniques in Bacterial Systematics.

[48] Yoon S.-H., Ha S.-M., Kwon S., Lim J., Kim Y., Seo H., Chun J. (2017). Introducing EzBioCloud: A taxonomically united database of 16S rRNA gene sequences and whole-genome assemblies. Int. J. Syst. Evol. Microbiol..

[49] Magoc T., Salzberg S.L. (2011). FLASH: Fast Length Adjustment of Short Reads to improve genome assemblies. Bioinformatics.

[50] Windowed Adaptive Trimming for Fastq Files Using Quality. https://github.com/najoshi/sickle.

[51] Bankevich A., Nurk S., Antipov D., Gurevich A.A., Dvorkin M., Kulikov A.S., Lesin V.M., Nikolenko S.I., Pham S., Prjibelski A.D. (2012). SPAdes: A new genome assembly algorithm and its applications to single-cell sequencing. J. Comput. Biol..

[52] Jain C., Rodriguez-R L.M., Phillippy A.M., Konstantinidis K.T., Aluru S. (2018). High throughput ANI analysis of 90K prokaryotic genomes reveals clear species boundaries. Nat. Commun..

[53] Meier-Kolthoff J.P., Auch A.F., Klenk H.-P., Göker M. (2013). Genome sequence-based species delimitation with confidence intervals and improved distance functions. BMC Bioinform..

[54] Meier-Kolthoff J.P., Carbasse J.S., Peinado-Olarte R.L., Göker M. (2022). TYGS and LPSN: A database tandem for fast and reliable genome-based classification and nomenclature of prokaryotes. Nucleic Acids Res..

[55] Tatusova T., Di Cuccio M., Badretdin A., Chetvernin V., Ciufo S., Li W. (2013). Prokaryotic genome annotation pipeline. The NCBI Handbook.

[56] Brettin T., Davis J.J., Disz T., Edwards R.A., Gerdes S., Olsen G.J., Olson R., Overbeek R., Parrello B., Pusch G.D. (2015). RASTtk: A modular and extensible implementation of the RAST algorithm for building custom annotation pipelines and annotating batches of genomes. Sci. Rep..

[57] Overbeek R., Olson R., Pusch G.D., Olsen G.J., Davis J.J., Disz T., Edwards R.A., Gerdes S., Parrello B., Shukla M. (2014). The SEED and the rapid annotation of microbial genomes using subsystems technology (RAST). Nucleic Acids Res..

[58] Grant J.R., Enns E., Marinier E., Mandal A., Herman E.K., Chen C., Graham M., Van Domselaar G., Stothard P. (2023). Proksee: In-depth characterization and visualization of bacterial genomes. Nucleic Acids Res..

[59] Kanehisa M., Sato Y., Morishima K. (2016). BlastKOALA and GhostKOALA: KEGG tools for functional characterization of genome and metagenome sequences. J. Mol. Biol..

[60] Wattam A.R., Davis J.J., Assaf R., Boisvert S., Brettin T., Bun C., Conrad N., Dietrich E.M., Disz T., Gabbard J.L. (2017). Improvements to PATRIC, the all-bacterial Bioinformatics Database and Analysis Resource Center. Nucleic Acids Res..

[61] Davis J.J., Gerdes S., Olsen G.J., Olson R., Pusch G.D., Shukla M., Vonstein V., Wattam A.R., Yoo H. (2016). PATtyFams: Protein families for the microbial genomes in the PATRIC Database. Front. Microbiol..

[62] Letunic I. (2016). and P. Bork, Interactive tree of life (iTOL) v3: An online tool for the display and annotation of phylogenetic and other trees. Nucleic Acids Res..

[63] National Center for Biotechnology Information. https://www.ncbi.nlm.nih.gov/genome/.

[64] KEGG PATHWAY Database. https://www.genome.jp/kegg/pathway.html.

[65] Harrison K.J., de Crécy-Lagard V., Zallot R. (2017). Gene Graphics: A genomic neighborhood data visualization web application. Bioinformatics.

[66] Andersen S.M., Johnsen K., Sørensen J., Nielsen P., Jacobsen C.S. (2000). *Pseudomonas frederiksbergensis* sp. nov., isolated from soil at a coal gasification site. Int. J. Syst. Evol. Microbiol..

[67] Zhang Y.Q., Li W.J., Kroppenstedt R.M., Kim C.J., Chen G.Z., Park D.J., Xu L.H., Jiang C.L. (2005). *Rhodococcus yunnanensis* sp. nov., a mesophilic actinobacterium isolated from forest soil. Int. J. Syst. Evol. Microbiol..

[68] Zhang D.C., Schumann P., Liu H.C., Xin Y.H., Zhou Y.G., Schinner F., Margesin R. (2010). *Arthrobacter alpinus* sp. nov., a psychrophilic bacterium isolated from alpine soil. Int. J. Syst. Evol. Microbiol..

[69] Dodor D.E., Tabatabai M.A. (2007). Arylamidase activity as an index of nitrogen mineralization in soils. Commun. Soil Sci. Plant Anal..

[70] Margesin R., Miteva V. (2011). Diversity and ecology of psychrophilic microorganisms. Res. Microbiol..

[71] Alori E.T., Glick B.R., Babalola O.O. (2017). Microbial phosphorus solubilization and its potential for use in sustainable agriculture. Front. Microbiol..

[72] Rizvi A., Ahmed B., Khan M.S., Umar S., Lee J. (2021). Psychrophilic bacterial phosphate-biofertilizers: A novel extremophile for sustainable crop production under cold environment. Microorganisms.

[73] Ruberto L., Vazquez S.C., Mac Cormack W.P. (2003). Effectiveness of the natural bacterial flora, biostimulation, and bioaugmentation on the bioremediation of a hydrocarbon-contaminated Antarctic soil. Int. Biodeterior. Biodegrad..

[74] Chaudhary D.K., Kim J. (2016). *Sphingomonas naphthae* sp. nov., isolated from oil-contaminated soil. Int. J. Syst. Evol. Microbiol..

[75] Chen L., Chen W.F., Xu Z.L., Li W., Zhang X.Y., Li W.J., Wang L. (2018). *Sphingomonas oleivorans* sp. nov., isolated from oil-contaminated soil. Int. J. Syst. Evol. Microbiol..

[76] Chun J., Oren A., Ventosa A., Christensen H., Arahal D.R., da Costa M.S., Rooney A.P., Yi H., Xu X.W., De Meyer S. (2018). Proposed minimal standards for the use of genome data for the taxonomy of prokaryotes. Int. J. Syst. Evol. Microbiol..

[77] Monisha T.R., Ismailsab M., Masarbo R., Nayak A.S., Karegoudar T.B. (2018). Degradation of cinnamic acid by a newly isolated bacterium *Stenotrophomonas* sp. TRMK2. 3 Biotech..

[78] Kertesz M.A., Kawasaki A., Timmis K.N. (2010). Hydrocarbon-Degrading Sphingomonads: *Sphingomonas*, *Sphingobium*, *Novosphingobium*, and *Sphingopyxis*. Handbook of Hydrocarbon and Lipid Microbiology.

[79] Oelschlagel M., Ruckert C., Kalinowski J., Schmidt G., Schlomann M., Tischler D. (2015). *Sphingopyxis fribergensis* sp. nov., a soil bacterium with the ability to degrade styrene and phenylacetic acid. Int. J. Syst. Evol. Microbiol..

[80] Whyte L.G., Schultz A., Beilen J.B., Luz A.P., Pellizari V., Labbé D., Greer C.W. (2002). Prevalence of alkane monooxygenase genes in Arctic and Antarctic hydrocarbon-contaminated and pristine soils. FEMS Microbiol. Ecol..

[81] Rodrigues D.F., Tiedje J.M. (2008). Coping with our cold planet. Appl. Environ. Microbiol..

