# Supplementary material for: Crude Oil Degradation in Temperatures Below the Freezing Point by Bacteria from Hydrocarbon-Contaminated Arctic Soils and the Genome Analysis of Sphingomonas sp. AR_OL41
Source: Microorganisms. 2023 Dec 30;12(1):79. doi: 10.3390/microorganisms12010079 (PMC10818417; doi:10.3390/microorganisms12010079)
Supplement: Supplementary file 1 [file microorganisms-12-00079-s001.zip › microorganisms-2798157-supplementary.pdf]

microorganisms

MDPI

Supplementary Materials

Crude Oil Degradation in Temperatures Below the Freezing Point by Bacteria from Hydrocarbon-Contaminated Arctic Soils and the Genome Analysis of *Sphingomonas* sp. AR_OL41

Ekaterina M. Semenova [1], Tatyana P. Tourova [1], Tamara L. Babich [1], Ekaterina Y. Logvinova [1], Diyana S. Sokolova [1], Nataliya G. Loiko [1], Vladimir A. Myazin [2,3,*], Maria V. Korneykova [2,3], Andrey V. Mardanov [4], and Tamara N. Nazina [1,*]

[1] Winogradsky Institute of Microbiology, Research Center of Biotechnology of the Russian Academy of Sciences, Moscow, 119071, Russia; semenova_inmi@mail.ru (E.M.S.); tptour@rambler.ru (T.P.T.); microb101@yandex.ru (T.L.B.); logvinovaekaterina@gmail.com (E.Y.L); sokolovadiyana@gmail.com (D.S.S.); loikonat@mail.ru (N.G.L.); nazina@inmi.ru (T.N.N.)

[2] Institute of North Industrial Ecology Problems - Subdivision of the Federal Research Centre "Kola Science Centre of Russian Academy of Science", 184209 Apatity, Russia; v.myazin@ksc.ru (V.A.M.)

[3] RUDN University, Agrarian and Technological Institute of the People's Friendship University of Russia, Moscow, 117198, Russia; korneykova.maria@mail.ru (M.V.K.)

[4] Institute of Bioengineering, Research Center of Biotechnology of the Russian Academy of Sciences, Moscow 119071, Russia; mardanov@biengi.ac.ru (A.V.M.)

* Correspondence: nazina@inmi.ru, Tel.: +7-499-135-0341 (T.N.N.); v.myazin@ksc.ru, Tel.: +7-815-557-97-71 (V.A.M.)

This file includes:

Figures S1 to S8

Tables S1 to S3

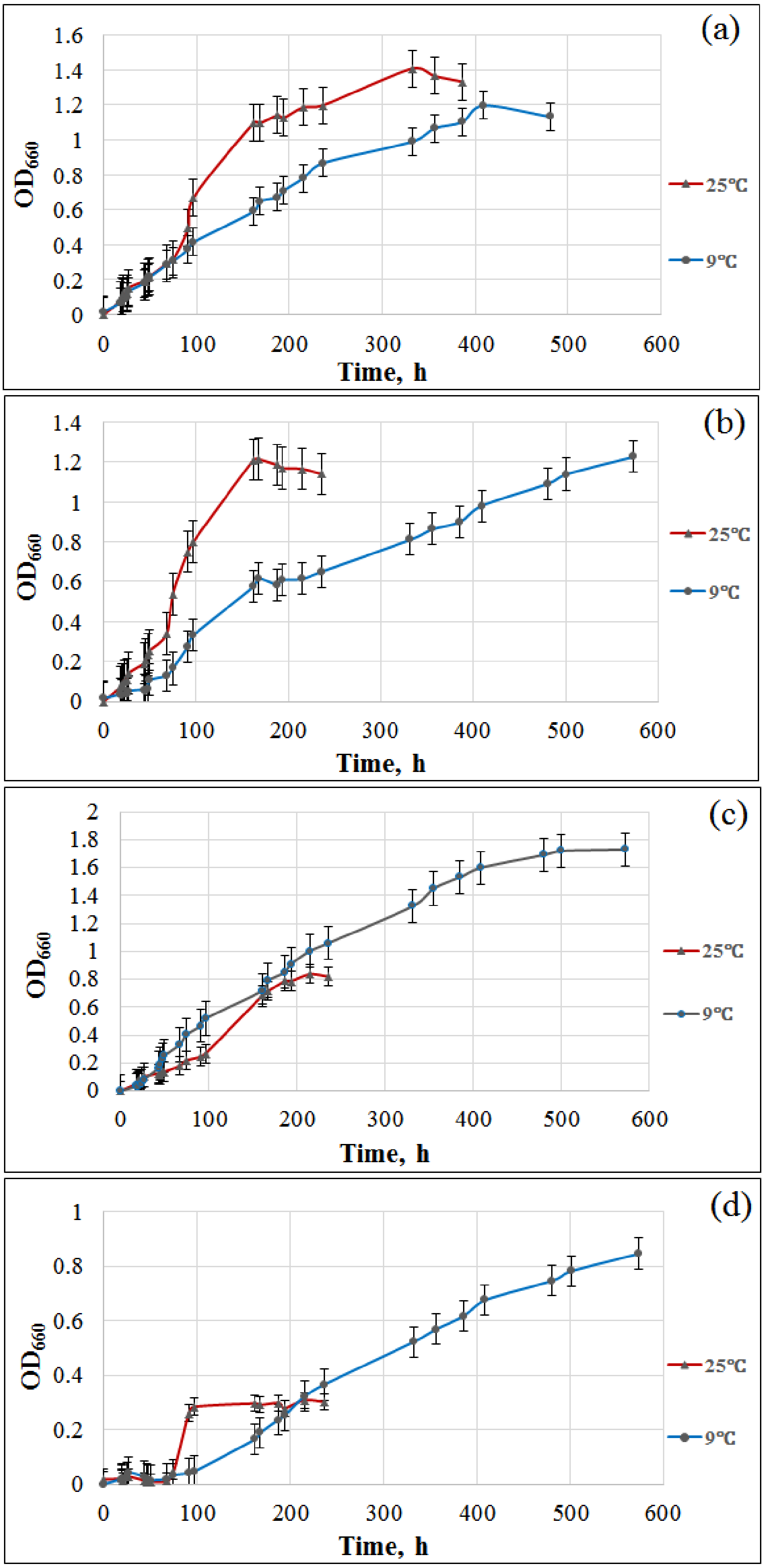

Figure S1. Growth curves of the strains *Pseudomonas frederiksbergensis* Ar-K7 (a), *Rhodococcus yunnanensis* Ar-K9 (b), *Arthrobacter alpinus* Ar-K10 (c), and *Sphingomonas* sp. AR_OL41 (d) in the TEG liquid medium at 9 °C and 25 °C.

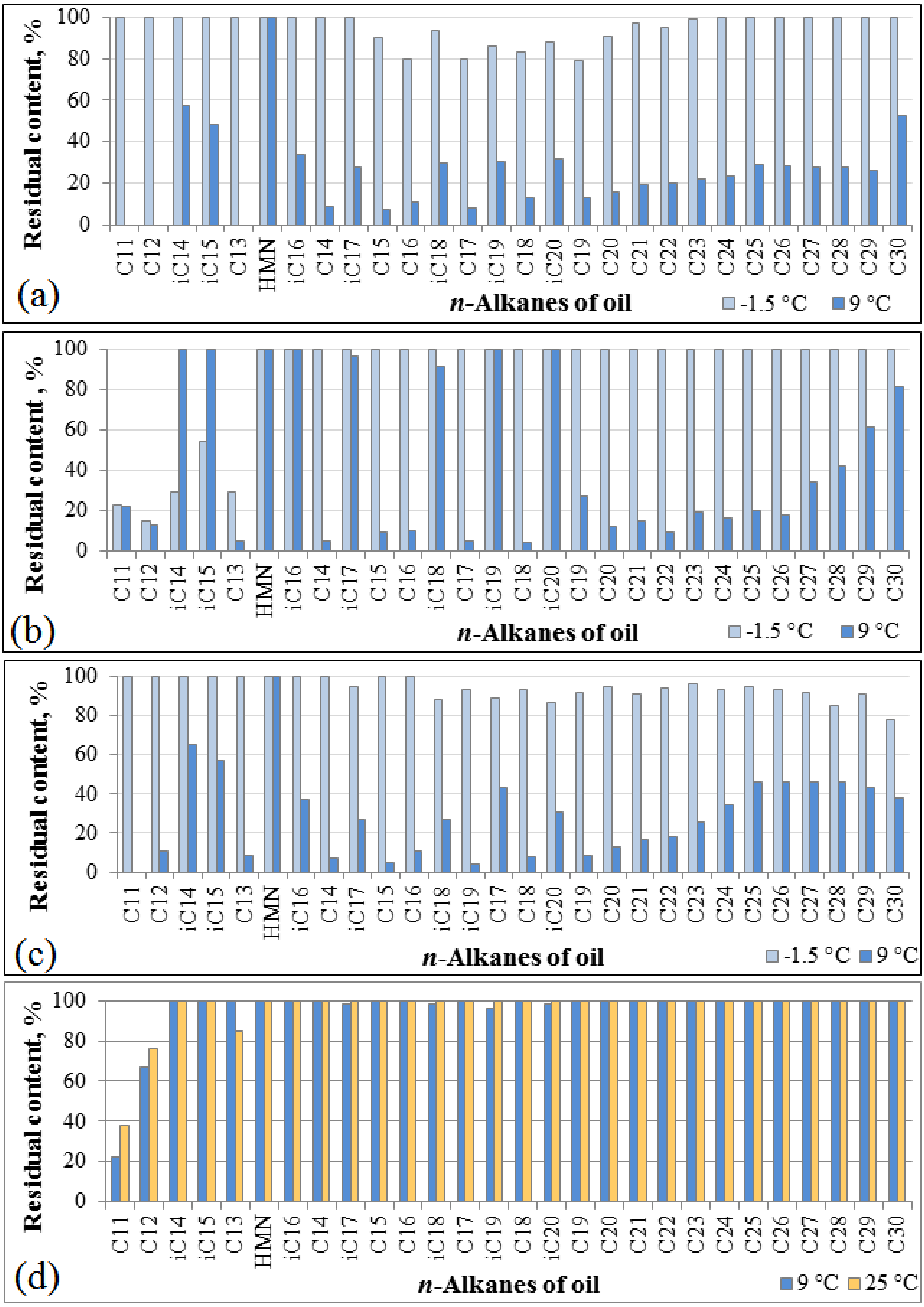

Figure S2. Residual content of *n*-alkanes in crude oil biodegraded by *Pseudomonas frederiksbergensis* Ar-K7 (a), *Rhodococcus yunnanensis* Ar-K9 (b), *Arthrobacter alpinus* Ar-K10 (c), and *Sphingomonas* sp. AR_OL41 (d) at –1.5 °C, 9 °C, and 25 °C.

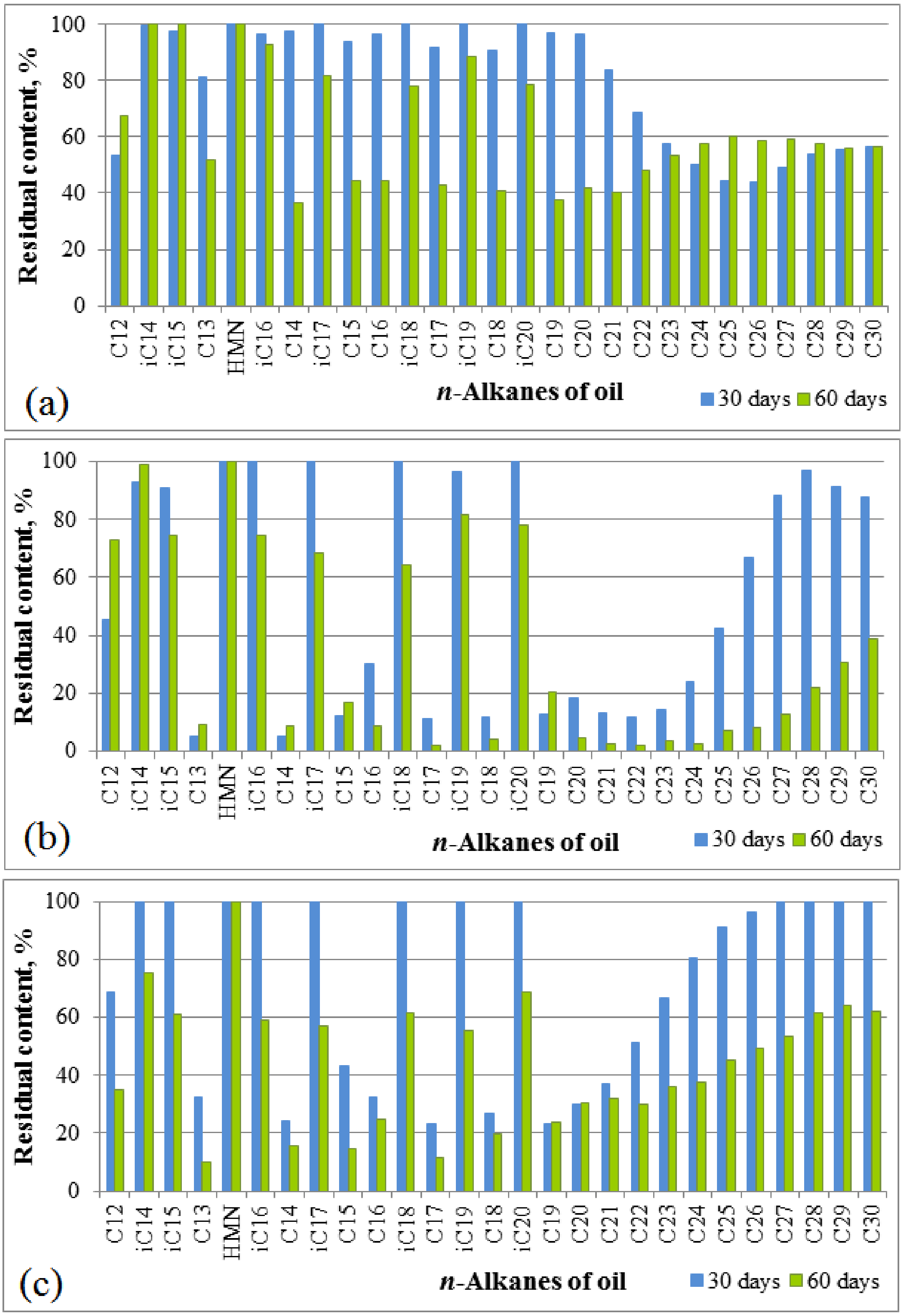

Figure S3. Residual content of *n*-alkanes (%) in crude oil biodegraded by *Pseudomonas frederiksbergensis* Ar-K7 (a), *Rhodococcus yunnanensis* Ar-K9 (b), and *Arthrobacter alpinus* Ar-K10 (c) during remediation of polluted sand.

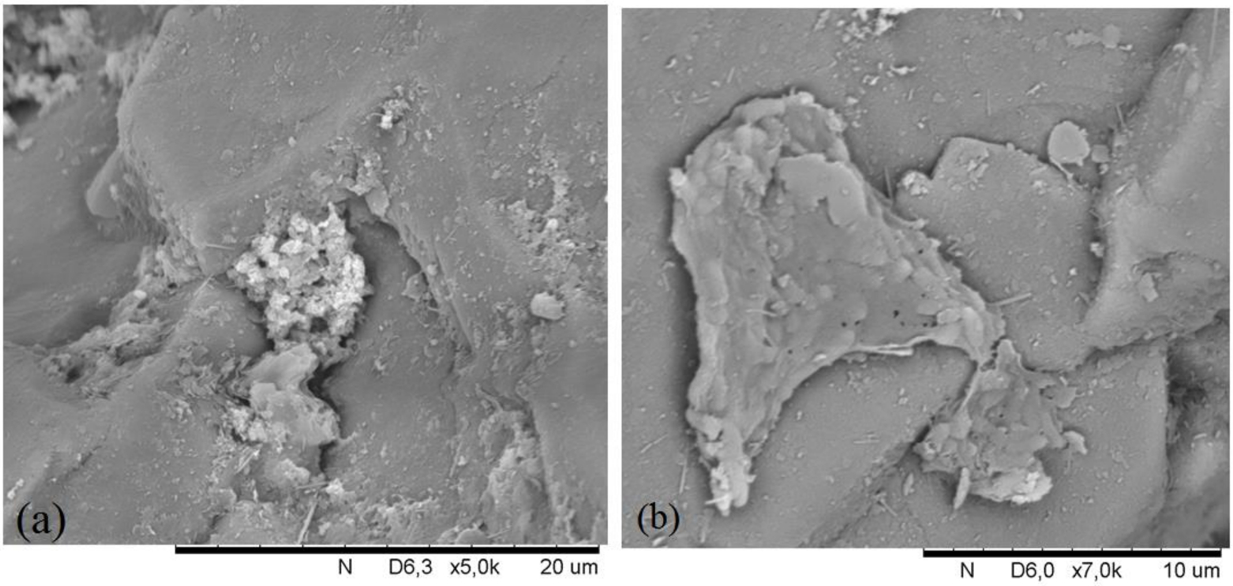

Figure S4. Scanning electron micrographs of biofilms of the strains *Pseudomonas frederiksbergensis* Ar-K7 (a) and *Rhodococcus yunnanensis* Ar-K9 (b) after 30 days of cultivation on oil-contaminated sand. The samples were examined under a scanning electron microscope TM3000 (Hitachi, Tokyo, Japan) with accelerating voltage 15 kV.

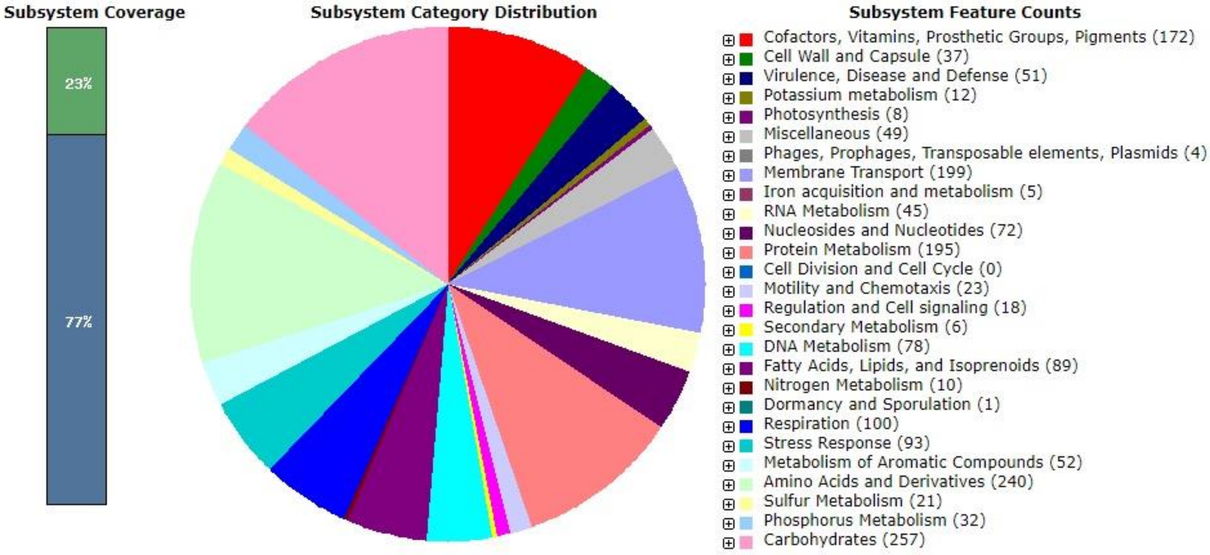

Figure S5. Subsystems of *Sphingomonas* sp. AR_OL41 based on RAST database.

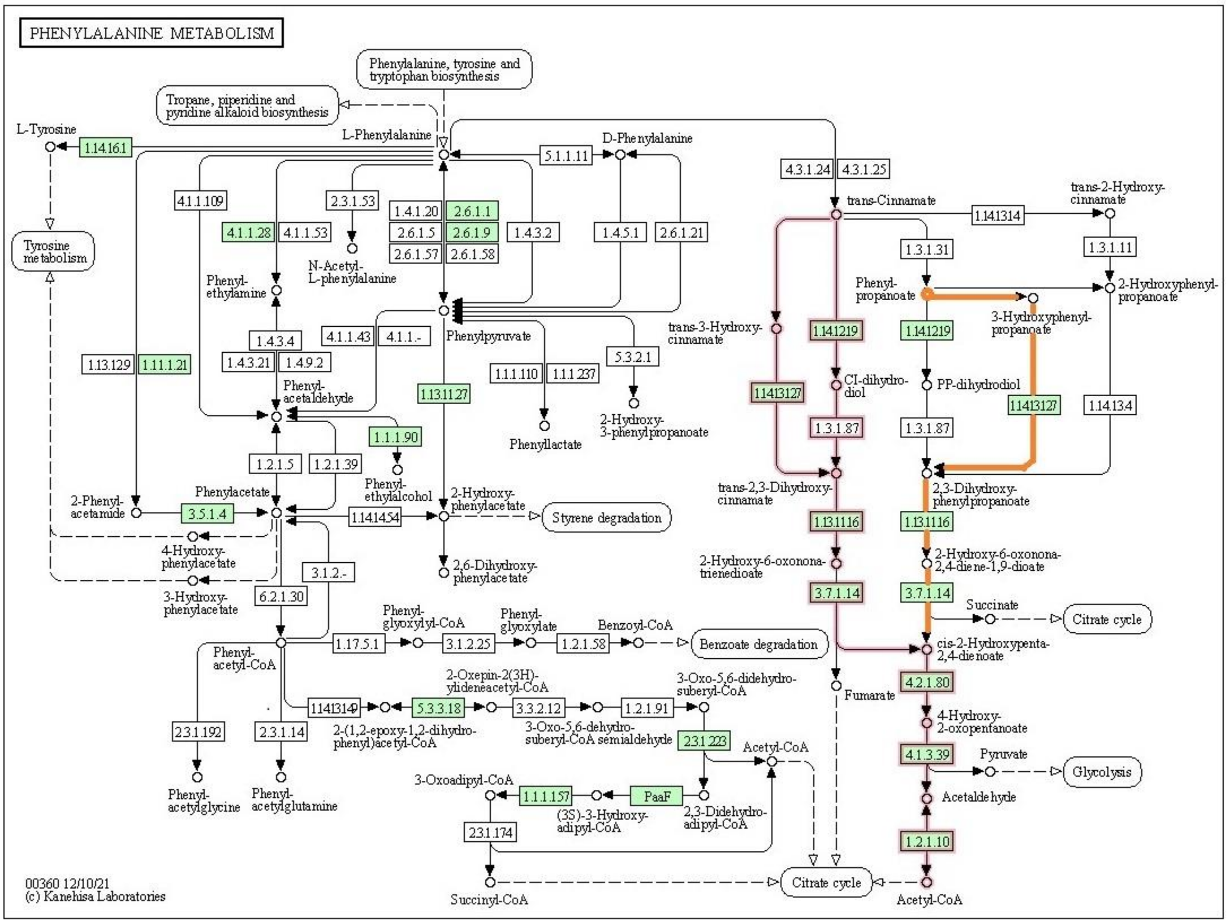

Figure S6. KEGG-map of phenylalanine metabolism (id 00360) with degradation modules of trans-cinnamic and phenylpropionic acids based on genome analysis of the strain *Sphingomonas* sp. AR_OL41.

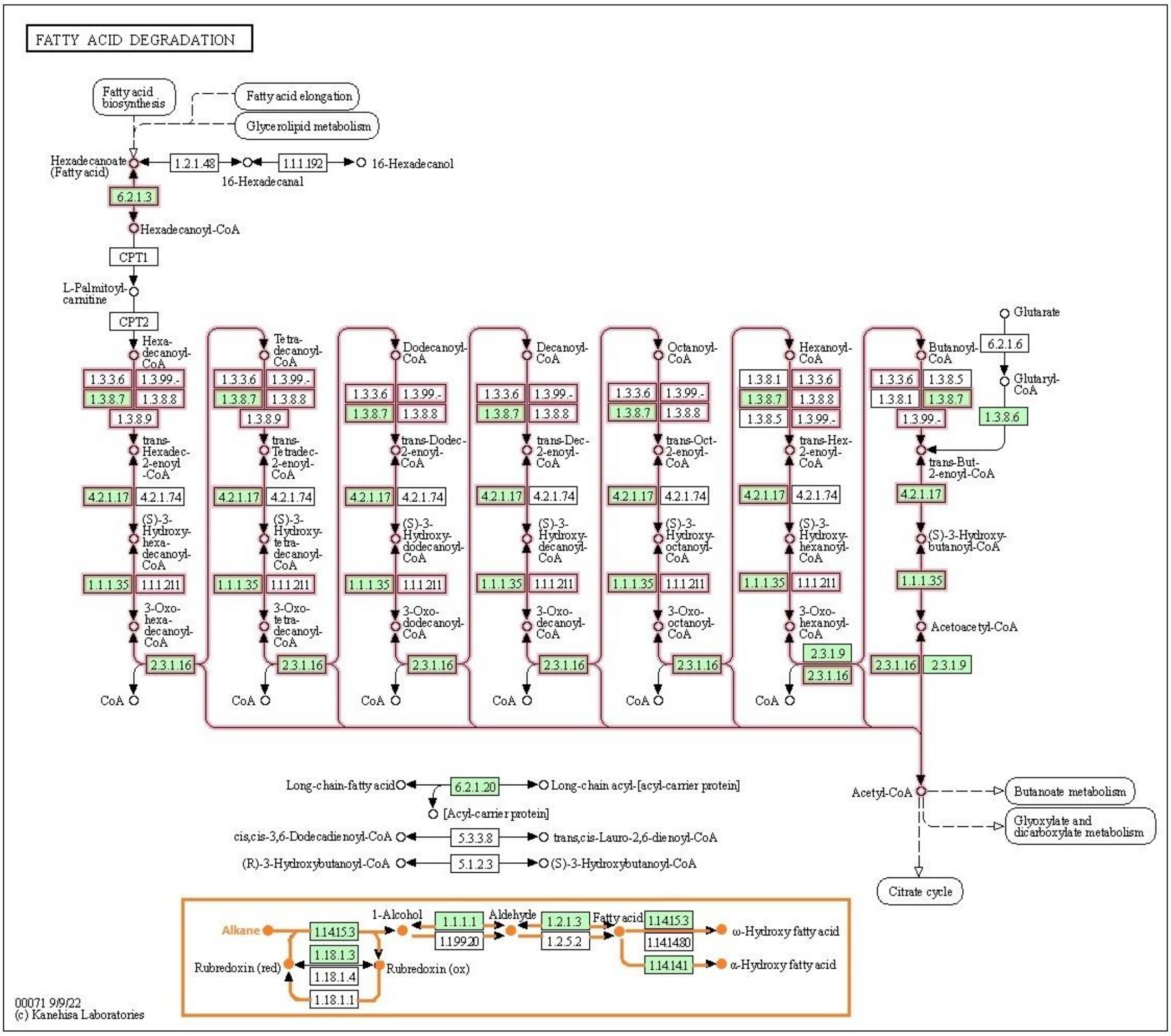

Figure S7. KEGG-map of degradation of fatty acids and *n*-alkanes (id 00071) based on genome analysis of the strain *Sphingomonas* sp. AR_OL41.

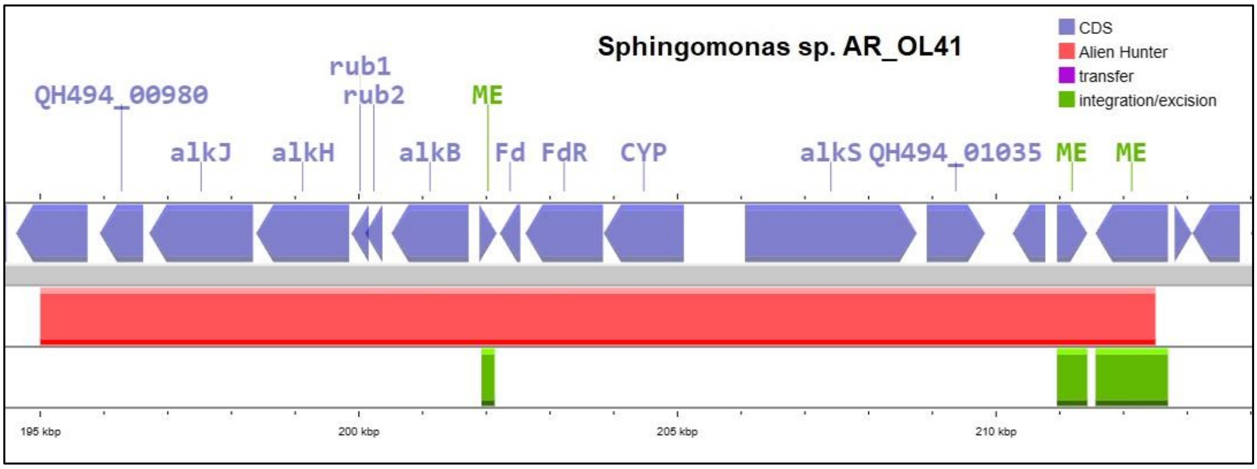

Figure S8. Localization of *n*-alkane degradation genes on the graphic map of the circular chromosome of the strain *Sphingomonas* sp. AR_OL41. The genes of mobile elements (ME) are highlighted in green. Designations: *alkB* – alkane-1 monooxygenase; *rub* – rubredoxin; *alkH* – aldehyde dehydrogenase; *alkJ* – alcohol dehydrogenase; Fd – ferredoxin; FdR – ferredoxin reductase; CYP – presumably cytochrome P450; *alkS* – transcriptional regulator; ME – proteins of mobile elements.

Table S1. Phenotypic characteristics of hydrocarbon-oxidizing bacteria *Pseudomonas frederiksbergensis* Ar-K7, *Rhodococcus yunnanensis* Ar-K9, *Arthrobacter alpinus* Ar-K10, and *Sphingomonas* sp. AR_OL41.

Characteristic	***Pseudomonas frederiksbergensis* Ar-K7**	***Rhodococcus yunnanensis* Ar-K9**	***Arthrobacter alpinus* Ar-K10**	***Sphingomonas* sp. AR_OL41**
Temperature range/ optimum, °C	–1.5…25/5	5…25/ 5–15	–1.5…25/ 10–25	9…25/20–25
NaCl range/optimum, % *w*/*v*	0–1.5/0.2	0–4/0.5	0–6/0–4	0–1.5/0
API® ZYM tests:				
Alkaline phosphatase	+	+	+	+
Esterase (C4)	+	W	+	+
Esterase lipase (C8)	+	+	+	+
Lipase (C14)	+	+	W	–
Leucine arylamidase	+	+	+	+
Valine arylamidase	+	+	+	+
Cystine arylamidase	+	+	+	+
Trypsin	–	+	+	–
α-Chymotrypsin	–	+	+	–
Acid phosphatase	+	+	+	+
Naphthol-AS-BI-phosphohydrolase	+	+	+	+
α-Galactosidase	–	–	+	–
ß-Galactosidase	–	–	+	–
ß-Glucuronidase	–	–	+	+
α-Glucosidase	–	–	+	+
ß-Glucosidase	–	+	+	+
α-Mannosidase	–	–	+	–
API® 20E tests:				
ß-Galactosidase	–	–	+	–
Arginine dihydrolase	+	–	–	–
Citrate utilization	+	–	+	–
Acid from glucose	+	+	–	–
Xylan	+	–	–	+
Chitin	+	+	+	+
Catalase	+	+	+	+
Oxidase	+	–	–	+

Results of the API® 20E tests for all studied strains were negative for lysine decarboxylase, ornithine decarboxylase, H_2S production, urease, tryptophane deaminase, indole production, acetoin production (Voges Proskauer), and gelatinase. In the API® ZYM tests, all four studied strains were negative for N-acetyl-ß-glucosaminidase and α-fucosidase.

Table S2. Physiological growth parameters of *Pseudomonas frederiksbergensis* Ar-K7, *Rhodococcus yunnanensis* Ar-K9, and *Arthrobacter alpinus* Ar-K10 on oil-contaminated sand.

Sample	0 days		30 days				60 days				Oil decrease, mg/100 g of sand
	Cells number per g of sand	Biofilm density, OD_{540}	Cells number per g of sand	Biofilm density, OD_{540}	pH	Acetate/ ethanol, mg/kg of sand	Cells number per g of sand	Biofilm density, OD_{540}	pH	Acetate/ ethanol, mg/kg of sand	
Ar-K7 (Control without oil)	10^7	-	10^7	0.32	7.7	81.6 / 11.0	10^8	0.24	8.1	20.0 / 0.8	-
Ar-K7	10^8	0.12	10^7	0.35	7.7	22.1 / 5.1	10^7	0.70	8.0	12.5 / 1.9	87±13
Ar-K9 (Control without oil)	10^3	-	0	0.15	7.8	19.4 / 5.7	0	0.14	7.8	23.0 / 6.4	-
Ar-K9	10^3	0.14	10^6	0.64	7.7	15.6 / 6.5	10^7	0.56	7.8	6.8 / 2.0	37±6
Ar-K10 (Control without oil)	10^7	-	10^6	0.20	7.8	18.1 / 5.3	10^6	0.14	7.8	8.1 / 0.7	-
Ar-K10	10^7	0.13	10^7	0.30	7.8	34.9 / 6.0	10^7	0.48	7.9	11.0 / 1.0	70±11

Table S3. Genes implicated in the adaptation to cold environments in the genome of *Sphingomonas* sp. AR_OL41.

Functional classification	Protein name	Gene Symbol	No. of genes	RefSeq Locus Tag
DNA replication	DNA gyrase subunit A (EC: 5.99.1.3)	*gyrA*	1	QH494_18230
	RecA protein	*gecA*	1	QH494_01255
	Chromosomal replication initiator protein DnaA	*dnaA*	1	QH494_20750
Nucleoid protein; DNA supercoiling	DNA-binding protein HU-beta		2	QH494_00455
				QH494_08860
RNA chaperones	Cold shock protein, CspA family		5	QH494_04405
				QH494_09435
				QH494_14005
				QH494_17445
				QH494_25730
Protein folding	Chaperone protein DnaK	*dnaK*	2	QH494_12850
				QH494_14160
	Chaperone protein DnaJ	*dnaJ*	1	QH494_12855
	Peptidyl-prolyl cis-trans isomerase (EC: 5.2.1.8)		3	QH494_11325
Protein biosynthesis	Translation initiation factor 1	*infA*	1	QH494_24975
	Ribosome-binding factor A	*rbfA*	1	QH494_27105
Unsaturation of membrane lipids	Fatty acid desaturase, type 2		2	QH494_04100
	Delta-9 fatty acid desaturase (EC: 1.14.19.1)		3	QH494_17510
				QH494_24800
				QH494_25035
Clustering-based subsystems	Exopolysaccharide biosynthesis protein		1	QH494_20245
Pyruvate metabolism II	Pyruvate dehydrogenase E1 component alpha subunit (EC: 1.2.4.1)	*pdhA*	2	QH494_02965
				QH494_02965
	Pyruvate dehydrogenase E1 component beta subunit (EC: 1.2.4.1)	*pdhB*	2	QH494_02960
				QH494_02960
	Pyruvate dehydrogenase E2 component (dihydrolipoyllysine-residue acetyltransferase) (EC: 2.3.1.12)	*pdhC*	1	QH494_06455
